# Fetal programming of the cardiac mitochondrial permeability transition pore in male offspring from hypoxic pregnancies

**DOI:** 10.1016/j.redox.2025.103975

**Published:** 2025-12-11

**Authors:** Kerri L.M. Smith, Philippe Pasdois, Mafalda Pires, Mitchell C. Lock, Gina L.J. Galli

**Affiliations:** a Faculty of Biology, Medicine and Health, University of Manchester, Manchester, UK; b Univ. Bordeaux, CNRS, IBGC, UMR 5095, Bordeaux, F-33000, France

**Keywords:** MPTP, Hypoxia, Fetal, Heart, ROS, Programming

## Abstract

A lack of oxygen during fetal development (fetal hypoxia) permanently alters the structure and function of the heart, leading to increased susceptibility to ischemia reperfusion (IR) injury in adulthood. However, the underlying cellular mechanisms are incompletely understood. In this study, we used a rat model to understand the role of calcium, reactive oxygen species and the mitochondrial permeability transition pore (MPTP) in programming IR sensitivity in offspring from hypoxic pregnancies. Pregnant Wistar rats were subjected to either ambient oxygen (∼21 %) throughout gestation, 13 % oxygen from gestational day 6–20, or 10.5 % oxygen from gestational day 15–20 (rat term ∼ 22 days). Offspring were raised to adulthood and hearts were subjected to *ex vivo* IR injury during Langendorff perfusion, whilst measuring ventricular pressure, intracellular calcium, oxidative stress and NAD(P)H autofluorescence. In addition, calcium retention capacity (CRC) and MPTP components were measured in isolated mitochondria, as well as basal H_2_O_2_ emission and electron transport system activity. Exposure to fetal hypoxia (10.5 % oxygen) increased IR sensitivity in adult offspring, demonstrated by increased diastolic pressure (*p* < 0.05), lipid peroxidation (*p* < 0.05), and an increased rate of NAD(P)H oxidation (*p* < 0.05) at reperfusion. This increased sensitivity to IR was associated with a decreased CRC (*p* < 0.01), increased basal H_2_O_2_ emission (*p* < 0.05) and decreased basal respiratory capacity linked to complex IV (*p* < 0.01). Additionally, both models of fetal hypoxia (13 % and 10.5 %) increased the abundance of the MPTP regulatory protein cyclophilin D in adult hearts (*p* < 0.01 and <0.001, respectively). Together, these data suggest that exposure to hypoxia during fetal development can programme MPTP calcium sensitivity by altering factors that modulate the pore (e.g. H_2_O_2_ emission, electron transport system activity, NAD(P)H oxidation and CypD content). These data could help to explain why individuals from hypoxic pregnancies are more susceptible to myocardial infarction, and other cardiovascular diseases.

## Graphical abstract

**Figure:**
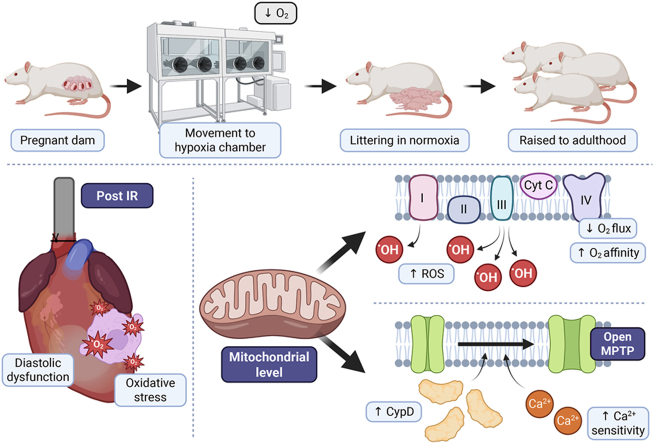
Created in BioRender. Smith, K. (2025) https://BioRender.com/w7k5b9u.

**Table:** Abbreviations

CRC	Calcium retention capacity
CS	Citrate synthase
CsA	Cyclosporine A
CypD	Cyclophilin D
ETS	Electron transport system
FGR	Fetal growth restriction
IR	Ischemia reperfusion
LVEDP	Left ventricular end diastolic pressure
MCU	Mitochondrial calcium uniporter
MPTP	Mitochondrial permeability transition pore
ROS	Reactive oxygen species
RPP	Rate pressure product
SUIT	Substrate-uncoupler-inhibitor-titration

## 1 Introduction

Fetal hypoxia is a common pregnancy complication, affecting up to 3 % of pregnancies in Europe [1]. Fetal hypoxia is prevalent among women living at high altitudes (over 6 % of the global population live over 1500 m above sea level [2]) and in maternal pathologies, such as preeclampsia (affecting 2–8 % pregnancies worldwide [3]) where disrupted placental perfusion leads to inadequate oxygen supply [4,5]. Other factors involved in the development of fetal hypoxia include placental insufficiency, anaemia, maternal undernutrition/smoking, fetal genetic factors, gestational diabetes, pollution exposure and umbilical cord compression/occlusion [4,6]. Exposure to hypoxia can mimic the placental pathologies associated with pregnancy disorders, as well as recapitulating both maternal and fetal symptoms that accompany these complex disorders, demonstrating that hypoxia itself may be the driving force in many complicated pregnancies [7,8]. Epidemiological studies have shown children from hypoxic pregnancies have higher systolic and diastolic blood pressure, increased relative cardiac wall thickness, reduced left ventricular end diastolic volume and left ventricular diastolic dysfunction [[9], [10], [11], [12], [13]]. These findings have been corroborated by animal models [[14], [15], [16]], which have also shown that fetal hypoxia sensitises the heart to ischemia-reperfusion (IR) injury [14,[17], [18], [19], [20], [21]]. This is particularly worrying, because children from hypoxic pregnancies are also at an increased risk of ischemic heart disease and myocardial infarction [22,23]. A deeper understanding of the underlying mechanisms driving IR sensitivity in these individuals could inform strategies to protect their heart from ischemic diseases.

One of the major drivers of IR injury is the mismanagement of cardiac myocyte calcium handling and opening of the mitochondrial permeability transition pore (MPTP). The MPTP is a multi-protein channel that opens in the inner mitochondrial membrane under conditions of matrix calcium overload and protein oxidation [24,25]. During ischemia, ATP-dependent ion transport fails, and cardiac myocyte intracellular calcium levels rise, leading to calcium overload [26]. When the affected area is reperfused, calcium enters the mitochondria and reactive oxygen species (ROS) increases, leading to MPTP opening. Sustained MPTP opening disrupts mitochondrial energy metabolism and triggers a series of events that culminate in necrotic and/or apoptotic cell death [27]. Although the exact molecular structure of the MPTP still remains controversial, the activity and expression of cyclophilin D (CypD), the adenine nucleotide translocator (ANT) and some subunits of the F_1_F_o_ ATP synthase are reported to influence the probability of MPTP opening [[27], [28], [29], [30], [31]]. Furthermore, in addition to calcium, several other MPTP activators have been identified, including high levels of matrix inorganic phosphate, a decreased NAD(P)H/NADP^+^ ratio and oxidative stress/protein oxidation [[32], [33], [34]]. On the other hand, divalent cations (e.g. Mg^2+^ and Ba^2+^), protons, adenine nucleotides and cyclosporine A (CsA) inhibit MPTP opening [27,35].

Importantly, we have recently shown that adult offspring from hypoxic pregnancies have abnormalities in cardiac myocyte excitation-contraction coupling, including prolonged calcium transients and higher levels of basal ROS production [20,36,37]. This phenotype is particularly dangerous for IR, because the calcium that accumulates during ischemia will be more difficult to remove during reperfusion and the ROS emission will be higher, leading to a greater probability of MPTP opening. Interestingly, fetal and adult heart tissue from hypoxic pregnancies also show differential expression of the gene that encodes cyclophilin D, and this appears to be regulated by differential gene methylation [38]. Lastly, the activity and expression of electron transport proteins (including the F_1_F_o_ ATP synthase) in fetal and adult hearts are altered by fetal hypoxia [36,39,40], as well as the expression of PKCε - a protein that is known to interact with and inhibit the MPTP [18,41,42]. Collectively, these studies suggest that aspects of the MPTP activation pathway are programmed by fetal hypoxia, which may help to explain why offspring from hypoxic pregnancies are more susceptible to IR injury.

In this study, we used a rat model to probe the mechanisms that promote IR sensitivity in offspring from hypoxic pregnancies. Firstly, we expected that offspring from hypoxic pregnancies would possess a cardiac mitochondrial phenotype that was more susceptible to MPTP opening, including heightened MPTP calcium sensitivity, increased basal H_2_O_2_ emission, differential protein expression of key MPTP components, and altered basal electron transport system (ETS) activity. Secondly, we hypothesised that *ex vivo* IR would lead to a more pronounced increase in intracellular calcium and ROS in adult offspring from hypoxic pregnancies, compared to controls. To our knowledge, this is the first study to investigate the possibility that the MPTP pathway can be programmed by intrauterine stress. Given its fundamental role in cell death in all cell types, intrauterine programming of the MPTP has clinical implications beyond fetal hypoxia and could be involved in the pathophysiology of a wide range of diseases.

## 2 Methods

### 2.1 Reagents

All chemicals and reagents were obtained from Sigma Aldrich unless otherwise stated.

### 2.2 Animal care

All procedures were carried out in accordance with The UK Animals (Scientific Procedures) Act, 1986. The ARRIVE guidelines were followed for reporting the use of animals in scientific experiments [43]. Local ethical approval was granted by The University of Manchester Animal Welfare Ethical and Review Board (PD7C22AA9). Female Wistar rats were purchased from Charles River (Charles River Laboratories, Saffron Walden, UK), either as virgins or time-mated. Rats were acclimatised for 1 week before time-mating or movement into the environmental chamber. During acclimatisation, rats were group-housed in individually ventilated cages under standard conditions (21 °C, 50 % humidity). Access to food (standard rat chow, Envigo, Indianapolis, USA) and water was provided ad libitum, and rats were subject to a regular light cycle of 12:12 h light/dark.

### 2.3 Animal model

For initial experiments concerning the mitochondrial phenotype, two different models of fetal hypoxia were used. The first was a “mild” model of early-onset hypoxia, where pregnant rats were subjected to inhalational hypoxia at an oxygen (O_2_) saturation of 13 % from gestational day (GD) 6–20. This model is relevant to human high-altitude pregnancies, and has little or no effect on maternal feeding [[44], [45], [46]], which allows us to identify the effects of fetal hypoxia alone, in the absence of nutritional stress. The second was a more “moderate” model of late-onset fetal hypoxia (10.5 % O_2_, GD 15–20), which is commonly used to recapitulate the effects of preeclampsia and placental insufficiency [8,47], as it is known to reduce both fetal O_2_ supply and nutrient availability (due to reduced maternal feeding). As the mitochondrial phenotype was repeatedly more affected by the late-onset model, the latter experiments involving *ex vivo* IR utilised this model only (10.5 % O_2_, GD 15–20).

Rats were singly housed and randomly assigned to 3 experimental groups: control (ambient O_2_ throughout gestation; 21 % O_2_, n = 12), mild hypoxia (13 % O_2,_ GD 6–20, n = 10), and moderate hypoxia (10.5 % O_2_, GD 15–20, n = 10). For hypoxia exposure, rat cages were moved to an environmental chamber (Coy Labs, USA) and the O_2_ concentration was steadily decreased to the desired concentration. Food and water consumption were measured daily, and maternal body weight was measured once prior to, during and after chamber incubation. On GD 20, all rats were returned to normoxia and allowed to litter (rat term ∼ 22 days). The day after littering, pups were weighed and culled by cervical dislocation to eight pups per dam to standardise maternal care. Hearts from spare neonates were frozen at −80 °C for Western blotting. Rats were weaned at 4 weeks, housed with same-sex litter mates and regularly weighed. Adult male rats were used for experimentation between four and seven months of age. Only one offspring per pregnancy was used for any one experiment. For all experiments with adult offspring, rats were killed via carbon dioxide inhalation and cervical dislocation.

### 2.4 Mitochondrial phenotype

#### 2.4.1 Isolation of mitochondria

Briefly, the heart was excised and atria and great vessels removed. Ventricular tissue was rinsed of blood several times using MSE buffer (in mmol/L, 5 MOPS, 2 NaEDTA, 70 sucrose, 220 mannitol, 0.1 % BSA, pH 7.35) and minced into small chunks. Tissue was then incubated with trypsin (#T0503, 6520 units/ml, in MSE minus BSA) for 10 min on ice and neutralised with a trypsin inhibitor (#T9128, 1 mg:1000 units of trypsin, in MSE). The tissue chunks were resuspended and homogenised using a glass tissue homogeniser. The resulting homogenate was centrifuged at 800×*g* (all centrifugation steps for 10 min at 4 °C), and the supernatant was filtered through gauze mesh before further centrifugation at 9000×*g*. The pellet was lightly rinsed with MSE buffer, and the supernatant was discarded. The rinsed pellet was resuspended and centrifuged at 9000×*g*. The resultant pellet was resuspended in MSE buffer (conc. 7.3 ± 0.2 mg/ml) and kept on ice. Following completion of experiments, the remaining suspension was aliquoted and frozen at −80 °C for future experiments.

#### 2.4.2 Calcium sensitivity of the MPTP

The calcium sensitivity of the MPTP was assessed with the well-established calcium retention capacity (CRC) assay, adapted from Bhosale and Duchen [48]. Firstly, a Bradford assay was used to determine the volume of mitochondria required for the CRC assay. Mitochondria (750 μg) were diluted in MSK buffer (in mmol/L, 75 mannitol, 25 sucrose, 5 KH_2_PO_4_, 20 Tris-HCl, 100 KCl, 0.1 % BSA, pH 7.4) and centrifuged at 9000×*g*. The pellet was resuspended in MSK + buffer (MSK buffer plus in mmol/L, 10 sodium succinate (#S2378), 0.001 rotenone (#R8875), 0.001 Fluo-5N [Invitrogen, #F14203]) at 500 μg/ml and kept on ice, protected from light. A 96-well plate was set up to include: buffer wells containing maximum calcium (100 μM) for autogain of spectrophotometer, mitochondria wells with no calcium additions, mitochondria wells with DMSO as vehicle control, mitochondria wells with Cyclosporine A (CsA [Tocris, #1101], 5 μM) to inhibit MPTP opening and buffer wells with calcium additions. All conditions were run in triplicate and averaged. Mitochondria were incubated with DMSO or CsA on ice for 10 min before commencing calcium additions. The spectrophotometer was maintained at 37 °C throughout the experiment. Calcium was rapidly added to the desired wells using a repeat pipettor, at a final concentration of 2 μM for the first five additions, then 5 μM for the next six additions, and 10 μM for the final six. Fluorescence was measured at excitation 485 nm/emission 528 nm every 10 s for 3 min (sampling rate of 1.58 samples/second), before the next addition of calcium. The MPTP was considered to have opened when there was no subsequent decrease in fluorescence following calcium addition, and the calcium concentration at this point was taken as the CRC. The area under the curve (AUC) was calculated using GraphPad Prism (v9.5.1).

#### 2.4.3 Activity of the MCU

The activity of the mitochondrial calcium uniporter (MCU) was estimated from calcium uptake rate during the CRC assay, which was determined from the decay of the fluorescence traces. Calcium additions two to six were selected for decay measurements to avoid artefacts. Background levels of fluorescence were recorded in the absence of calcium, and maximum calcium was recorded as the fluorescence in the presence of 100 μM calcium without mitochondria. GraphPad Prism (v9.5.1) was used to calculate the decay time (tau) of each calcium trace through non-linear regression. Results for these experiments are shown in Supplementary Figure 1.

#### 2.4.4 Protein abundance of cyclophilin D and ATP synthase

The abundance of MPTP components Cyclophilin D and the ATP synthase (subunits α and c) were measured in neonatal and adult hearts using Western Blotting. These proteins were chosen as they are critically involved in mitochondrial permeability transition regulation [31,[49], [50], [51]]. For adults, protein expression was determined in isolated mitochondria after use in the Oroboros experiments (see below). For neonates, whole hearts were collected from one-day old pups and suspended in RIPA buffer containing a protease and phosphatase inhibitor cocktail (#PPC1010) to create a 10 % homogenate. Homogenisation occurred in a Precellys Evolution, for six 20-s bursts at 6800 RPM. Homogenate was centrifuged at 1000×*g* for 10 min at 4 °C, and supernatant collected and stored at −80 °C. Protein concentration was measured using a DC protein assay (Bio-Rad).

Protein (either isolated cardiac mitochondria from adult tissue or whole ventricular homogenate from neonatal tissue) was diluted in RIPA buffer, denatured and loaded onto a 4–12 % Bis-Tris gel. For CypD, 10 μg was loaded for adult samples and 20 μg loaded for neonatal samples. For ATP synthase subunits, 1 μg was loaded for adult samples and 2 μg loaded for neonatal samples. Following electrophoresis, proteins were transferred to a nitrocellulose membrane and blocked using 5 % milk in TBS-T. Blots were incubated with primary antibodies (CypD [1:1000 dilution] – ab110324 anti-cyclophilin F, Abcam; ATP synthase subunit α [1:1000 dilution] – ab14748, anti-ATP5a, Abcam; ATP synthase subunit c [1:2000 dilution] – ab181243, anti-ATP synthase C, Abcam) overnight at 4 °C, before secondary antibody incubation (IRDye® 680RD goat anti-mouse for CypD and ATP synthase α subunit, IRDye® 680RD goat anti-rabbit for ATP synthase c subunit [both 1:20 000 dilution]) at room temperature for 1 h. Protein abundance was visualised by fluorescence (Licor-Odyssey CLx), and normalised to total protein (membrane stained using Revert 700 total protein stain, Licor) and an internal control (sample) for inter-membrane normalisation. All blots were repeated in triplicate and results averaged. Blots were analysed using Image Studio (v5.2). Original uncropped images of all Western Blot membranes can be found in Supplementary Figure 2.

#### 2.4.5 Gene expression of cyclophilin D and ATP synthase

RNA was extracted from 30 to 50 mg frozen left ventricular tissue using QIAzol lysis reagent (QIAgen, #79306) and QIAgen RNeasy mini purification columns, as per manufacturers guidelines (QIAgen). RNA was quantified using a NanoDrop Lite Spectrophotometer (ThermoFisher), with measurements taken at 260 and 280 nm. All samples produced a 260/280 nm ratio between 1.95 and 2.1 and therefore were acceptable quality for downstream qPCR. cDNA was synthesised using SuperScript™ IV Reverse Transcriptase (Invitrogen, #15307696), using 1 μg of total RNA with random hexamers, dNTP mix, DTT and SuperScript IV in a total volume of 20 μl. Reverse transcription was carried out using a C1000 thermocycler (Biorad), according to manufacturer's guidelines. Controls were set up to test for reagent or genomic DNA contamination, by removing RNA transcript or SuperScript from the reagent mix respectively.

Reference genes were determined from a panel of candidates using geNorm (part of qbase + software). For normalisation, three stable reference genes (*Rpl32, Rpl4* and *Polr2a*) were run simultaneously with target genes (*Atp5f1a, Atp5mc1 and Ppif*) (Supplementary Table 1). Primers were designed and validated for specificity via melt curve analysis. Relative expression of target genes was assessed using Power SYBR Green PCR Master Mix (Thermo, #4367659), in a final volume of 10 μl on a ViiA7 Fast Real-time PCR system (Applied Biosystems). Each well contained 7 μl SYBR Green Master Mix, 1 μl each of forward and reverse primer (final primer concentration 10 μM) and 1 μl of diluted cDNA (30 ng). Target and reference genes for each sample were run in triplicate. The abundance of each gene relative to the abundance of the reference genes was calculated using DataAssist 3.0 analysis software and expressed as mRNA mean normalised expression (MNE). Results for these experiments are shown in Supplementary Figure 3.

#### 2.4.6 Basal mitochondrial ETS activity and mitochondrial content

Basal electron transport system (ETS) activity and H_2_O_2_ emission were measured simultaneously in isolated ventricular mitochondria with an Oroboros Oxygraph O2K (Oroboros Instruments, Innsbruck, Austria). A substrate-uncoupler-inhibitor-titration (SUIT) protocol was utilised to sequentially examine different respiratory states.

An isolated mitochondrial suspension (2 μL, 14.5 ± 0.5 μg) was added to each chamber, followed by the substrates pyruvate (#P2256, 6.25 mM), malate (#M1000, 2 mM) and glutamate (#G1626, 10 mM), to achieve LEAK respiratory state through complex I in the absence of adenylates (LEAK_CI,n/adenylates_). ADP (#A5285, 250 μM) was then added to activate OXPHOS with complex I substrates (OXPHOS_CI_), and respiration was left to occur until ADP was depleted to achieve LEAK state in presence of adenylates (LEAK_CI,w/adenylates_). Succinate (10 mM) was then added (LEAK_CI + CII,w/adenylates_), followed by a further ADP (250 μM) addition to measure the additive effects of complex II substrates on OXPHOS (OXPHOS_CI + CII_). Mitochondria were then uncoupled through the addition of carbonyl cyanide-4-(trifluoromethoxy)phenylhydrazone (FCCP, #C2920), in order to assess maximum electron transfer capacity (ETC_CI + CII_). FCCP was titrated in steps to a final concentration of 0.75–3 μM, until additions failed to cause a subsequent increase in respiration rate. The complex I inhibitor rotenone (0.15 μM) was then added to assess ETC_CII_ with CII substrates only. Next, antimycin A (#A8674, 12.5 μM), the complex III inhibitor was added to block the electron transport pathway and measure residual nonmitochondrial O_2_ consumption (ROX). For complex IV activity determination, the electron donor N,N,N′,N′-tetramethyl-*p*-phenylenediamine (TMPD, #T3134, 0.5 mM) was added, together with ascorbate (#A4034, 2 mM) to prevent TMPD auto-oxidation. Finally, the CIV inhibitor sodium azide (#S2002, 50 mM) was added, to measure background nonmitochondrial O_2_ consumption following TMPD addition. Values at each respiratory state were normalised to protein concentration of the mitochondrial suspension.

To assess O_2_ affinity of complex IV, mitochondria (21.8 ± 0.8 μg) were added to an Oroboros chamber and supplied with substrates for complex I and II activity - glutamate (10 mM), malate (2 mM), pyruvate (6.25 mM) and succinate (10 mM). ADP was added at a saturating concentration (2.5 mM) and respiration allowed to occur until all O_2_ in the chamber was depleted. Anoxia was maintained for 10 min before chambers were re-oxygenated. A Michaelis-Menten curve was fit to the O_2_ flux trace, and K_m_ calculated using non-linear regression in GraphPad Prism (v9.5.1).

In a separate chamber, mitochondrial quality was assessed through addition of exogenous cytochrome *c* (#C2506, 10 μM) with percentage increase in respiration measured as proxy for mitochondrial outer membrane damage. The largest increase was 17 %, with an average increase of 13 ± 1 %, demonstrating good preservation of the mitochondrial outer membrane. For these experiments, oligomycin (#O4876, 25 nM) was also added to measure LEAK with oligomycin. Quality control parameters are shown in Supplementary Table 2.

Citrate synthase (CS) activity was measured to assess mitochondrial content using a spectrophotometer. Frozen left ventricle (50 mg) was homogenised in sucrose buffer (in mmol/L, 20 Tris base, 40 KCl, 2 EGTA, 250 sucrose), and centrifuged at 800×*g* at 4 °C for 10 min. Supernatant was diluted in CS activity buffer (100 μM 5,5′-dithiobis-(2-nitrobenzoic acid) [DTNB, #D218200], 300 μM acetyl coenzyme-A (#A2056), 100 mM Tris, 0.1 % Triton-X-100). Oxaloacetate (#O4126, 0.5 mM) was added to the samples, and absorbance measured repeatedly for 10 min at 412 nm to determine V_max_. CS activity values were normalised to wet weight of ventricular tissue.

#### 2.4.7 Basal H_2_O_2_ emission

Hydrogen peroxide (H_2_O_2_) emission was measured simultaneously with ETS activity (see above) through the addition of horseradish peroxidase (HRP, #P8250) (1 U/mL), superoxide dismutase (SOD, #S8160) (5 U/mL) and Amplex UltraRed (Invitrogen, #A36006, 10 μM) to each chamber, prior to mitochondrial addition. H_2_O_2_ causes oxidation of Amplex UltraRed to resorufin, with HRP as a catalyst, while SOD converts extramitochondrial superoxide (O_2_-) to H_2_O_2_. Amplex UltraRed fluorescence was measured through excitation at 563 nm and emission at 587 nm. For each experiment, an H_2_O_2_ internal calibration was carried out by adding two additions of H_2_O_2_ (40 μM) to achieve a chamber concentration of 0.1 and 0.2 μM. Values of H_2_O_2_ emission were normalised to oxygen consumption (see above), except for ROX which was normalised to protein concentration. Original traces of both oxygen consumption and H2O2 production are shown in Supplementary Figures 4 and 5.

### 2.5 Intracellular calcium and ROS during ischemia/reperfusion

#### 2.5.1 Ex vivo IR model

The Langendorff preparation was used to simultaneously measure ventricular pressure, intracellular calcium, NAD(P)H and oxidative stress during IR. Previous research has consistently demonstrated that fetal hypoxia increases cardiac sensitivity to IR injury (a summary of these findings can be found in Supplementary Table 3). In this study, we chose a brief period of ischemia (15 min) and reperfusion (30 min) to avoid extensive injury or ventricular fibrillation, which can interfere with fluorescent imaging. Although both hypoxia models (13 % and 10.5 %) have previously been shown to increase IR sensitivity, we used the 10.5 % model in this series of experiments because the phenotype tends to be more pronounced [18,19], and we repeatedly observed a more severe mitochondrial phenotype using the 10.5 % model.

The experimental set up is shown in Supplementary Figure 6. The heart was excised and immediately placed into ice-cold Krebs-Henseleit (KH) buffer (in mmol/L, 119 NaCl, 25 NaHCO_3_, 4 KCl, 1.2 KH_2_PO_4_, 1 MgCl_2_, 1.8 CaCl_2_, 10 glucose, gassed with 95 % O_2_/5 % CO_2_ [pH 7.4]) containing heparin (Wockhardt, #PL 29831/0105, 1000 units in 15 mL). The aorta was rapidly cannulated, and the coronary arteries were flushed *via* the aorta with roughly 5 mL KH containing a further 500 units of heparin. The cannula was attached to a Langendorff apparatus maintained at 37 °C and set at a constant pressure of 30–40 mmHg, with blood allowed to clear from the heart for 2–3 min via retrograde perfusion. Any excess tissue was trimmed away from the heart, and the left atrial appendage was removed to allow for balloon insertion. After 3 min, pressure was increased to 70 mmHg and a latex balloon (AD Instruments) was inserted into the left ventricle and inflated with water to achieve a left ventricular end diastolic pressure (LVEDP) of ∼5–10 mmHg. Heart rate (HR), left ventricular pressure and perfusion pressure were measured throughout the experiment using PowerLab with LabChart software (v8, AD Instruments). Example traces of left ventricular pressure are shown in Supplementary Figure 7. Left ventricular developed pressure (LVDP) was calculated as the difference between left ventricular systolic pressure (LVSP) and LVEDP. To calculate cardiac work, HR was multiplied by LVDP to generate the rate pressure product (RPP). A minimum RPP of 20 000 was required at the end of the baseline period for the experiment to continue. The heart was lowered into a custom-made water-jacketed glass chamber (Radnoti, Dublin, Ireland) and maintained at 37 °C for the duration of perfusion. Following a baseline period of 20 min, flow to the heart was stopped to induce ischemia. The ischemic period lasted for 15 min before buffer flow to the heart was restored. Coronary flow rate was measured at regular intervals during the experiment, and the effluent was collected at baseline and at 5-min post reperfusion. Following 30 min reperfusion, the heart was removed from the system, weighed, and the left ventricular tissue was flash-frozen and stored at −80 °C for further analysis (see below).

#### 2.5.2 Intracellular calcium and NAD(P)H measurements during IR

Bespoke epicardial fluorescence equipment designed by Cairn Research Ltd (Faversham, Kent) was used to measure intracellular calcium and NAD(P)H levels during the IR protocol. Autofluorescence of the epicardial heart tissue was measured to assess NAD(P)H redox level (and therefore the metabolic state of the tissue), and the fluorescent indicator Indo-1 AM (Invitrogen, #I1203) was used to determine intracellular calcium concentration.

Protocols were adapted from Andrienko et al. [52]. In brief, excitation light was directed to the heart via a bespoke probe that fitted through the glass jacketed chamber so it was positioned roughly 5 mm away from the left ventricular wall at an angle of approximately 30°, below the left atria (see Supplementary Figure 6A). The probe also collected emission light, splitting the beam through a multi-position filter wheel onto a photomultiplier (PMT). The filter wheels were set to rotate at 4 Hz, and fluorescence was captured for a 30 s period every 3 min, with longer capture periods for the transitions into ischemia and upon reperfusion. When capturing calcium transients during optimisation of dye loading and concentration, filter wheels were set to rotate at 50 Hz. When not capturing, the fluorescent light was switched off to prevent dye bleaching and tissue light damage. For all experiments, fluorescence light intensity, PMT voltage and gain were kept constant. Photomultiplier signals were integrated and displayed using WinEDR software (University of Strathclyde).

NAD(P)H was measured in the *ex vivo* heart by measuring cardiac autofluorescence (emission peak ∼ 440–470 nm). The heart was excited at 340 nm and the emission light was collected at 485 nm. The t = 0.5 of NAD(P)H increase and half-life of NAD(P)H decrease was calculated using non-linear regression of the fluorescence signals during IR. In a separate set of experiments, intracellular calcium in the heart was measured with 2 μM Indo-1 AM (Invitrogen), which was added to the KH buffer (supplemented with 1 mM probenecid (#P8761) to prevent dye leakage). For Indo-1 loading, hearts were initially perfused with KH buffer using a constant flow method achieving a perfusion pressure of ∼30–40 mmHg. Once blood had cleared and the latex balloon had been inserted and inflated (∼5 min), background fluorescence was measured for 5–6 min. Indo-1 was then added to the KH buffer (containing 0.01 % Pluronic™ F-127 (#P2443) in DMSO) and recirculated with perfusate reoxygenation. Indo-1 loading occurred for 30 min before perfusion was gradually (across 1–2 min) changed to a constant pressure method, with a pressure of ∼70 mmHg. A 20-min washout period for dye de-esterification was employed before the IR challenge commenced. The heart was excited at 340 nm, and emission light was collected at 405 and 485 nm. To control for autofluorescence artefacts, fluorescence signals were measured at 405 and 485 nm in unloaded hearts under identical conditions and settings. These signals were averaged (for each experimental group) and subtracted from the Indo-1 405 and 485 nm signals individually, before a ratio was calculated (405 nm/485 nm). Indo-1 loading was considered successful if the fluorescence signal after washout was at least 3-fold greater than background fluorescence in both wavelengths. A timeline of the Indo-1 loading protocol is shown in Supplementary Figure 6D, and images of a rat heart before and after Indo-1 loading are shown in Supplementary Figure 6B and 6C respectively.

### 2.6 Cell damage and oxidative stress

The effluent and frozen ventricular tissue recovered from reperfusion were analysed for markers of cell damage. Firstly, creatine kinase (CK) activity of the effluent was measured using Creatine Kinase Activity Assay Kit (#MAK116), according to kit instructions (limit of detection 30 mU/mL). CK activity was determined against a blank and a calibrator solution, calculated for effluent collected at baseline and 5-min post reperfusion, then normalised to baseline activity. Oxidative stress was measured both pre- and post-IR by utilising a TBARS assay (thiobarbituric acid [TBA] reactive substance) assay, which measures the levels of malondialdehyde, a by-product of cellular lipid peroxidation. Ventricular tissue, collected either at baseline or following the IR challenge, was briefly thawed and ∼50 mg homogenised at 4 °C in ice-cold 10 mM phosphate buffer (pH 7.2), containing 200 μM butylated hydroxytoluene (#W218405) to prevent artificial oxidative stress from tissue homogenisation. The resulting homogenates were added to a TBA solution (0.375 % w/v TBA (Fluorochem, #036438), 15 % w/v trichloroacetic acid (Fisher, #T-3000-53), 0.25 N hydrochloric acid) in a 1:2 ratio, mixed and incubated at 95 °C for 30 min. Samples were then cooled on ice for 10 min, before addition of 1-butanol (#281549) at a 2:1 ratio. Samples were centrifuged at 15000×*g* for 3 min, and the top layer containing the malondialdehyde-TBA adduct was collected. Absorbance was measured at 532 nm in a plate reader and malondialdehyde concentrations were determined against a standard curve of 1,1,3,3-tetraethoxypropane, and normalised to protein concentration.

### 2.7 Statistical analysis

Where possible, data files were coded so that analysis was carried out blind to experimental group. All statistical analysis was carried out on GraphPad Prism v9.5.1. For analysis where there were two independent factors, a two-way analysis of variance (ANOVA) was used with Šídák's post-hoc test. For remaining analysis, statistical testing was decided on number of groups to be tested and whether data was normally distributed (ND). Data sets were tested for normality using both a Shapiro-Wilk and Kolmogorov-Smirnov test. If ND in both tests and comparing two experimental groups, a two-tailed *t*-test was used. If ND and three experimental groups, a one-way ANOVA with Šídák's post-hoc test was used. If not ND and two experimental groups, a Mann-Whitney test was used. If not ND and three experimental groups, a Kruskal-Wallis with Dunn's post-hoc test was used. The statistical test for each experiment can be found in the associated figure legend. In both graphs and text, data is represented as mean ± SEM. A *p* value < 0.05 was considered statistically significant and *p* values < 0.1 were included on graphs and within the results section for clarity.

## 3 Results

### 3.1 Maternal and offspring biometry

**Fig. 1:**
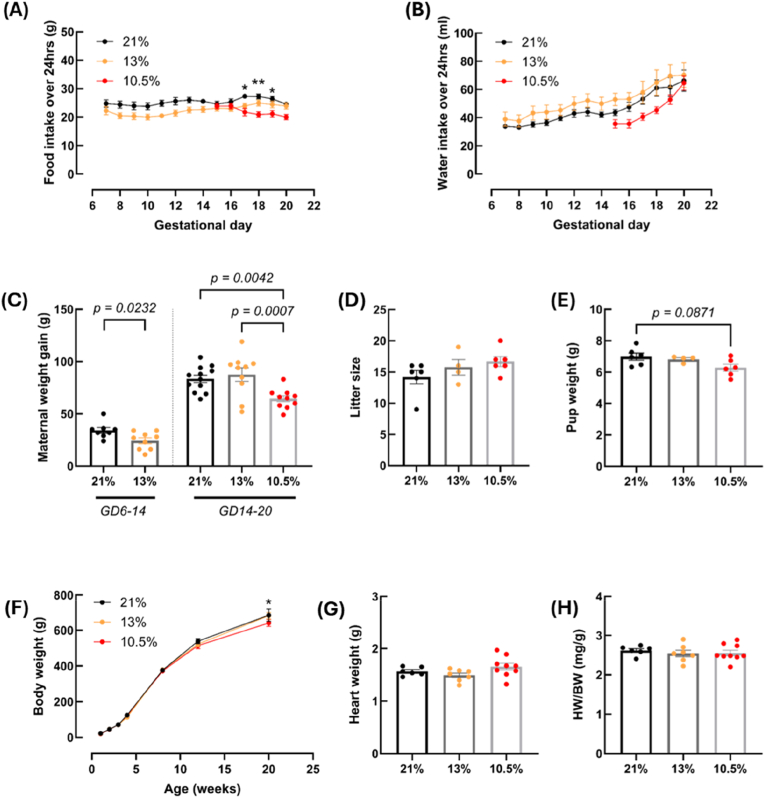
**Parameters for pregnant dams and offspring subjected to fetal hypoxia (GD 6-20 for control [21 % O_2_] and 13 % O_2_, GD 15**–**20 for 10.5 % O_2_).** Food and water intake of the dam were measured each day during hypoxia exposure (days 6–20 for controls and 13 % O_2_ groups, days 15–20 for 10.5 % O_2_ group) (A and B). Dam weight was measured at multiple points during hypoxia exposure and used to calculate weight gain from GD 6-14 (controls and 13 % O_2_ groups) and GD 14-20 (all groups) (C). Litter size was measured 1 day after littering (D). Pups were individually weighed aged 1 day (E) and then at regular intervals until adulthood (F). Heart weight was measured at experimentation (G) and used to calculate heart/body weight ratio (H). *For food intake and adult body weight, ∗ shows SD between 21 % and 10.5 % (∗ < 0.05, ∗∗ < 0.01). For offspring parameters, data points represent litter averages. For maternal data, n = 12 (21 %), n = 10 (13 %), n = 10 (10.5 %). For offspring data, n = 6 litters (21 %), n = 4-7 litters (13 %), n = 6-9 litters (10.5 %). HW = heart weight, BW = body weight. Data is presented as mean ± SEM. Significance was tested using two-way ANOVA for (A), (B) and (F), one-way ANOVA for (C) and (G), and Kruskal-Wallis for (D), (E) and (H).*

Similar to previous findings [53], maternal food intake was not affected by 13 % O_2_ exposure, but it was significantly reduced by 10.5 % O_2_ from day 17–19 of gestation (*p* = 0.0114 gestational day (GD) 17, *p* = 0.0011 GD 18 and *p* = 0.0243 GD 19) (Fig. 1A). Neither of the hypoxia models affected water intake of the pregnant dam (Fig. 1B), but they both lowered maternal weight gain at different gestational time points (Fig. 1C). For the 13 % hypoxia model, the initial weight gain (GD 6–14) was reduced (*p* = 0.0232), but late-stage weight gain (GD 14–20) was comparable. Late-stage weight gain in the 10.5 % hypoxia model was reduced compared to both controls (*p* = 0.0007) and 13 % hypoxia (*p* = 0.0042).

No differences were seen in litter size between experimental groups (Fig. 1D). Additionally, neither 10.5 % nor 13 % hypoxia caused fetal growth restriction (FGR) of pups, although 10.5 % approached significance (*p* = 0.0871) (Fig. 1E). Offspring body weight remained comparable between experimental groups up to three months of age, but at 20 weeks individuals from the 10.5 % hypoxia group weighed less than controls (*p* = 0.0261) (Fig. 1F). At four months, heart weight and heart weight normalised to body weight was comparable between all three groups (Fig. 1G and H).

### 3.2 MPTP calcium sensitivity

**Fig. 2:**
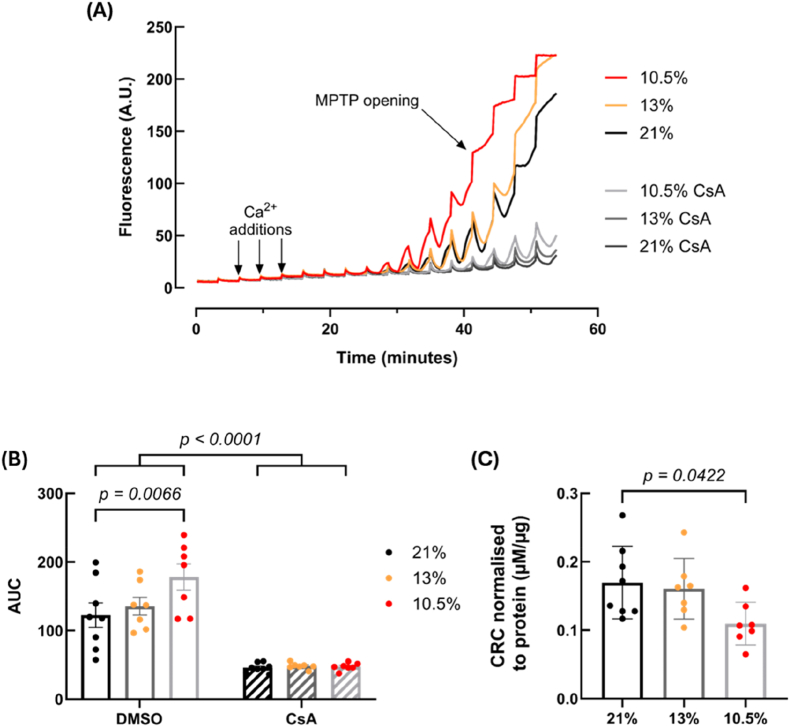
**Fetal hypoxia (GD 15**–**20, 10.5 % O_2_) decreases the calcium retention capacity of heart mitochondria compared to controls (21 % O_2_).** Representative traces of isolated mitochondria subject to repeated calcium additions (labelled), both with and without CsA (A). Traces were used to calculate the AUC after a set number of calcium additions (B), and used to calculate the point of MPTP opening, taken as the CRC for that animal and normalised to mitochondrial protein concentration (C). *n = 8 (21 %), 7 (13 %) and 7 (10.5 %). AU = arbitrary units, AUC = area under curve, CsA = cyclosporine A, CRC = calcium retention capacity, MPTP = mitochondrial permeability transition pore. Data is presented as mean ± SEM. Significance was tested using two-way ANOVA for (B) and one-way ANOVA for (C).*

We first determined the inherent calcium sensitivity of the MPTP using the well-established calcium retention capacity assay. Mitochondria responded as expected, initially taking up the extramitochondrial calcium, observed by a reduction in fluorescence of the extramitochondrial calcium-sensitive fluorescent dye, after each calcium addition (Fig. 2A). In the 10.5 % hypoxia group, there was a decrease in the number of calcium additions required to induce MPTP opening, compared to controls (decrease in the area under the calcium curves [*p* = 0.0066]) (Fig. 2B) and therefore a decrease in the calcium concentration required to reach CRC (*p* = 0.0422) (Fig. 2C). No significant differences were seen when comparing the 13 % hypoxia group to either the 10.5 % group or the controls. Cyclosporine A (CsA) was effective at inhibiting MPTP opening in all groups (*p* < 0.0001, Fig. 2A and B). No differences were seen when comparing the CsA treated wells among experimental groups (Fig. 2B). The rate of calcium uptake into mitochondria was measured from the decay of the calcium curves in the CRC assay (Supplementary Figure 1). Normalised representative traces are shown in Supplementary Figure 1B. No differences were observed between the experimental groups, either for individual calcium additions or when averaged across calcium additions 2–6 (Supplementary Figure 1C). This result suggests that mitochondrial calcium uptake was comparable between the three groups, suggesting similar calcium uptake rate efficiency.

### 3.3 MPTP molecular components

**Fig. 3:**
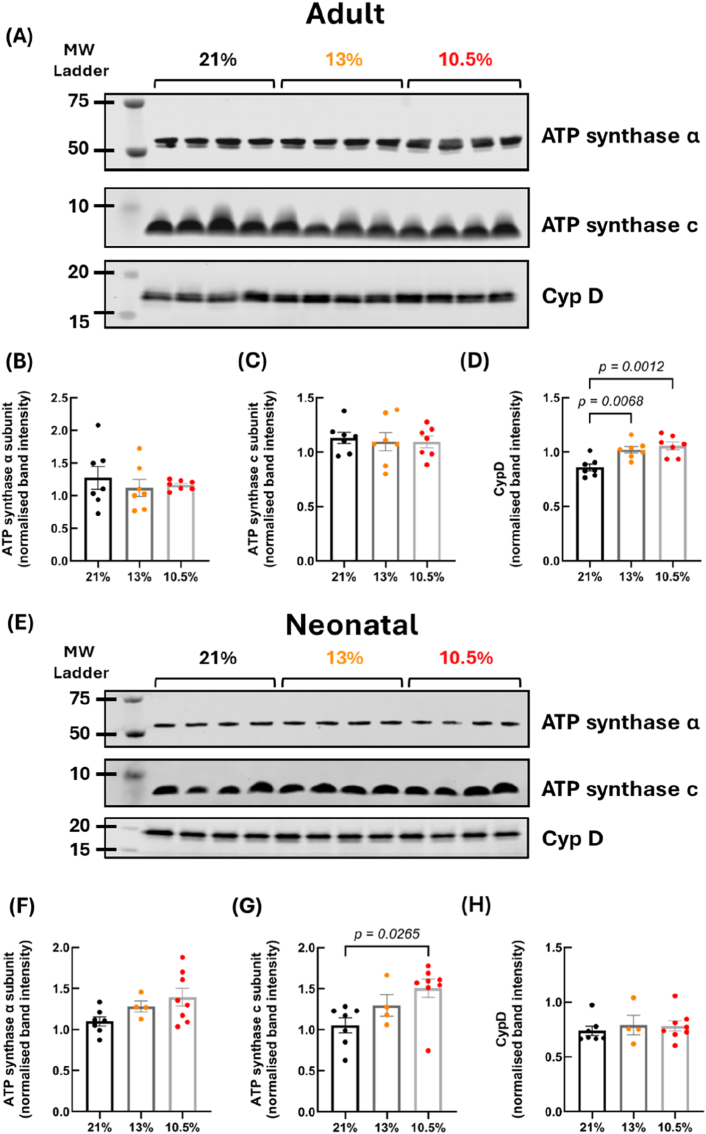
**Fetal hypoxia (GD 6**–**20, 13 % O_2_ and GD 15**–**20, 10.5 % O_2_) increases protein abundance of CypD in adult mitochondria.** Representative blots for ATP synthase subunits α and c and CypD in adult cardiac mitochondria (A) and neonatal ventricular homogenate (E). Band intensity was normalised to protein and to an internal sample control for ATP synthase α subunit (B) and (F), ATP synthase c subunit (C) and (G), and CypD (D) and (H). *For adult, n = 7 (21 %), 7 (13 %) and 7 (10.5 %). For neonatal, n = 7 (21 %), 4 (13 %) and 8 (10.5 %). CypD = cyclophilin D, MW = molecular weight. Data is presented as mean ± SEM. Significance was tested using one-way ANOVA for (B), (C) and (D), and Kruskal-Wallis for (F), (G) and (H).*

To investigate the molecular mechanisms driving the increase in CRC sensitivity, we looked at several known MPTP modulators. Firstly, we measured the protein expression of two proteins that are thought to be MPTP components; CypD and ATP synthase (subunits α and c) (Fig. 3A and E). Protein abundance of CypD was increased in both the 13 % and 10.5 % hypoxic groups compared to controls (*p* = 0.0068 and 0.0012 respectively) (Fig. 3D). No difference was observed in the abundance of either ATP synthase subunit in adult mitochondria (Fig. 3B and C). mRNA expression was also measured in adult left ventricular tissue, with no changes observed in the expression of *ATPf1a* (ATP synthase alpha subunit), *ATP5mc1* (ATP synthase c subunit) or *Ppif* (CypD) following fetal hypoxia (Supplementary Figure 3).

To determine whether these changes to MPTP components and CRC sensitivity were present from birth or developed with age, we also assessed the abundance of these MPTP components in homogenates from neonatal hearts. We found that the abundance of the ATP synthase c subunit was increased in the neonatal 10.5 % hypoxic group (*p* = 0.0265) (Fig. 3F and G); however, the α subunit and CypD were comparable across all three groups (Fig. 3H).

### 3.4 Mitochondrial H_2_O_2_ emission

**Fig. 4:**
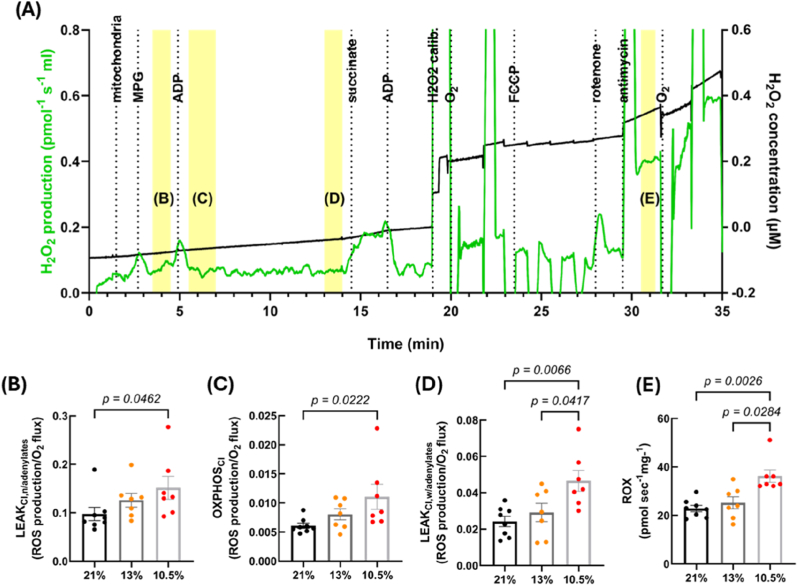
**Fetal hypoxia (GD 15**–**20, 10.5 % O_2_) increases H_2_O_2_ emission (normalised to oxidative capacity) at multiple states of the electron transport system.** Mitochondrial H_2_O_2_ emission and oxidative capacity was measured in mitochondria isolated from hearts of rats subject to control (21 % O_2_) or hypoxic (either GD 6-20, 13 % O_2_ or GD 15-20, 10.5 % O_2_) development. Each panel shows a different respiratory state, which relates to the highlighted region on the example Oroboros trace (A). Leak respiration with CI substrates without adenylates (B), oxidative phosphorylation with CI substrates (C), leak respiration with CI substrates with adenylates (D), and residual O_2_ consumption (E). *n = 8 (21 %), 7 (13 %) and 7 (10.5 %). CI = complex I, CII = complex II, MPG = malate, pyruvate and glutamate, ADP = adenosine diphosphate, FCCP = carbonyl cyanide-4-(trifluoromethoxy)phenylhydrazone. All states were normalised to oxidative capacity, except for (E) as mitochondrial O*_*2*_*consumption at this state is zero. Data is presented as mean ± SEM. Significance was tested using Kruskal-Wallis for all parameters except for (D) which used one-way ANOVA.*

When H_2_O_2_ emission was normalised to mitochondrial O_2_ consumption, the 10.5 % hypoxia group had significantly higher levels in multiple respiratory states, compared to controls: LEAK_CI,n/adenylates_ (*p* = 0.0462) (Fig. 4B), OXPHOS_CI_ (*p* = 0.0222) (Fig. 4C), LEAK_CI,w/adenylates_ (*p* = 0.0066) (Fig. 4D) and ROX (normalised to protein concentration) (p = 0.0026) (Fig. 4E); and at the latter two states when comparing 13 % and 10.5 % hypoxia (p = 0.0417 and 0.0284). No difference was seen in normalised H_2_O_2_ in any of the other states (Supplementary Figure 4). A typical Oroboros trace is shown in Fig. 4A, with the respiratory states that showed significant differences highlighted in yellow.

### 3.5 Electron transport system activity

Electron transport system activity was also measured in isolated mitochondria. The quality of ventricular mitochondria was excellent, as demonstrated by minimal percentage increases in respiration with addition of exogenous cytochrome *c* (13 % ± 1), suggesting mitochondrial membranes were intact [54] (Supplementary Table 2). Additionally, high and consistent respiratory control ratios (RCR) following inhibition with oligomycin (6.7 ± 0.2) were observed across isolations (Supplementary Table 2).

**Fig. 5:**
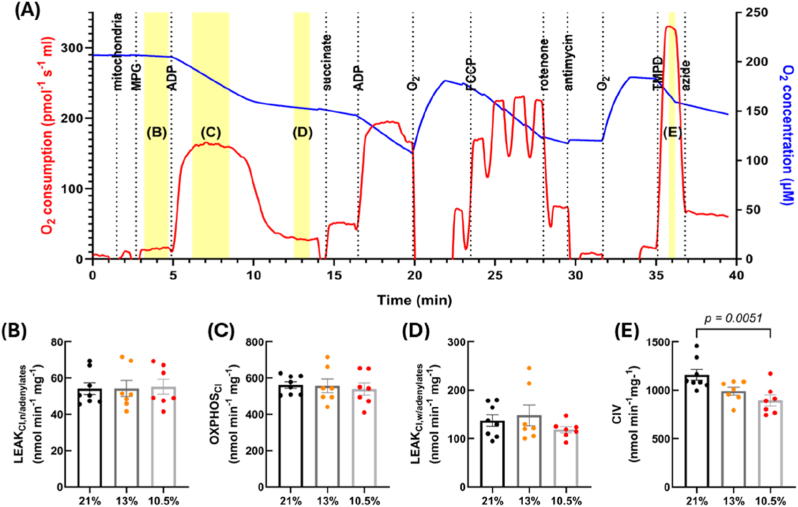
**Fetal hypoxia (GD 15**–**20, 10.5 % O_2_) reduces oxidative capacity at complex IV of the electron transport system.** Mitochondrial oxidative capacity was measured in mitochondria isolated from hearts of rats subject to control (21 %) or hypoxic (either 13 % GD 6-20 or 10.5 % GD 15-20) development. Each panel shows a different respiratory state, that relates to the highlighted region on the above Oroboros trace (A). Leak respiration with CI substrates without adenylates (B), oxidative phosphorylation with CI substrates (C), leak respiration with CI substrates with adenylates (D), and electron donation to CIV (E). *n = 8 (21 %), 7 (13 %) and 7 (10.5 %). CI = complex I, CII = complex II, CIV = complex IV, MPG = malate, pyruvate and glutamate, ADP = adenosine diphosphate, FCCP = carbonyl cyanide-4-(trifluoromethoxy)phenylhydrazone, TMPD = N,N,N,N-tetramethyl-p-phenylenediamine. Data is presented as mean ± SEM. Significance was tested using one-way ANOVA for (C) and (E), and Kruskal-Wallis for (B) and (D).*

**Fig. 6:**
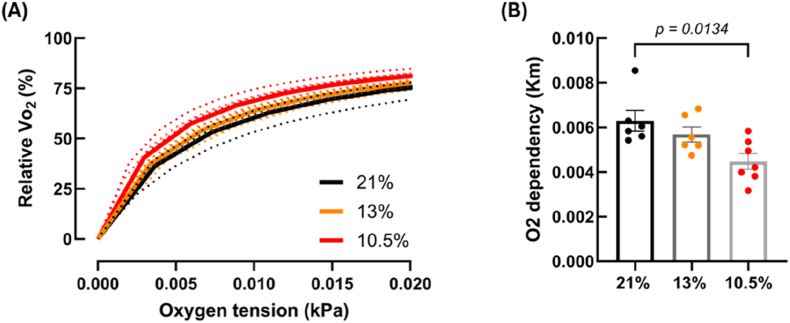
**Fetal hypoxia (GD 15**–**20, 10.5 % O_2_) increases O_2_ affinity of complex IV.** Traces showing respiration rate (Vo_2_) as a function of O_2_ concentration. Representative lines are shown in solid line, whilst individual curves for each experiment are shown as dotted lines (A). The Km value (O_2_ tension when V_O2_ equals 50) is shown in (B). *n = 6 (21 %) and 7 (10.5 %). Data is presented as mean ± SEM. Significance was tested using a Mann-Whitney test.*

To determine the effects of fetal hypoxia on mitochondrial ETS activity, O_2_ consumption was measured alongside H_2_O_2_ emission. Oxygen consumption was decreased following addition of complex IV (CIV) substrates in the 10.5 % hypoxic group compared to controls (*p* = 0.0051) (Fig. 5E), whilst all other respiratory states were comparable. The corresponding respiratory states that showed an increase in H_2_O_2_ emission are shown in Fig. 5B–D, whilst the remaining states are shown in Supplementary Figure 5. No differences were observed when looking at the 13 % hypoxic group. As complex IV function was reduced in the 10.5 % hypoxic group, we also looked at the O_2_ dependence of this complex. We found that the 10.5 % hypoxic group had a lower K_m_ and therefore a higher affinity for O_2_ compared to controls (*p* = 0.0047) (Fig. 6A and B). However, there were no differences in coupling efficiency or P/E ratio (index of limitation by the phosphorylation system) between the experimental groups, suggesting that mitochondrial efficiency and excess capacity was similar (Supplementary Table 2). Furthermore, total citrate synthase activity in ventricular homogenates was also comparable between the three experimental groups, suggestive of no changes in mitochondrial content (Supplementary Table 2).

### 3.6 Ventricular performance during IR

**Fig. 7:**
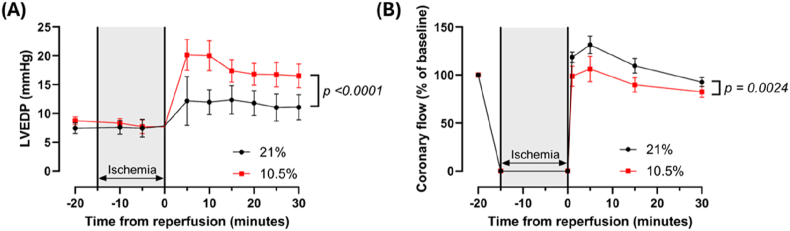
**Adult rats subjected to fetal hypoxia (GD 15**–**20, 10.5 % O_2_) show decreased post-ischemic recovery compared to controls (21 % O_2_).** LVEDP was calculated twice at baseline and averaged, and then at 5-min intervals throughout 15 min ischemia and 30 min reperfusion (A). Coronary flow was measured twice at baseline and averaged, and then at 1 min, 5 min, 15 min and 30 min post reperfusion (B). *n = 6. For LVEDP, ∗ = p < 0.05. LVEDP = left ventricular end diastolic pressure. Data is presented as mean ± SEM. Significance was tested using two-way ANOVA.*

As the 10.5 % hypoxic group had shown a more severe phenotype when investigating mitochondrial function, the remaining experiments investigating the *ex vivo* IR phenotype were carried out using this model only. In agreement with previous work (Supplementary Table 3), we found that the 10.5 % hypoxic group were more sensitive to IR than controls. Following 15 min of ischemia, LVEDP was significantly higher in the 10.5 % hypoxic group than controls across the 30-min reperfusion period (*p* < 0.0001) (Fig. 7A). Coronary flow was also reduced in the 10.5 % hypoxic group across the 30-min reperfusion period (*p* = 0.0024) (Fig. 7B). As expected with this length of ischemia [45,46], RPP was not significantly changed at reperfusion compared to baseline, and no differences in RPP were seen between the two experimental groups (Supplementary Table 4).

### 3.7 Intracellular calcium, NAD(P)H levels and oxidative stress during IR

**Fig. 8:**
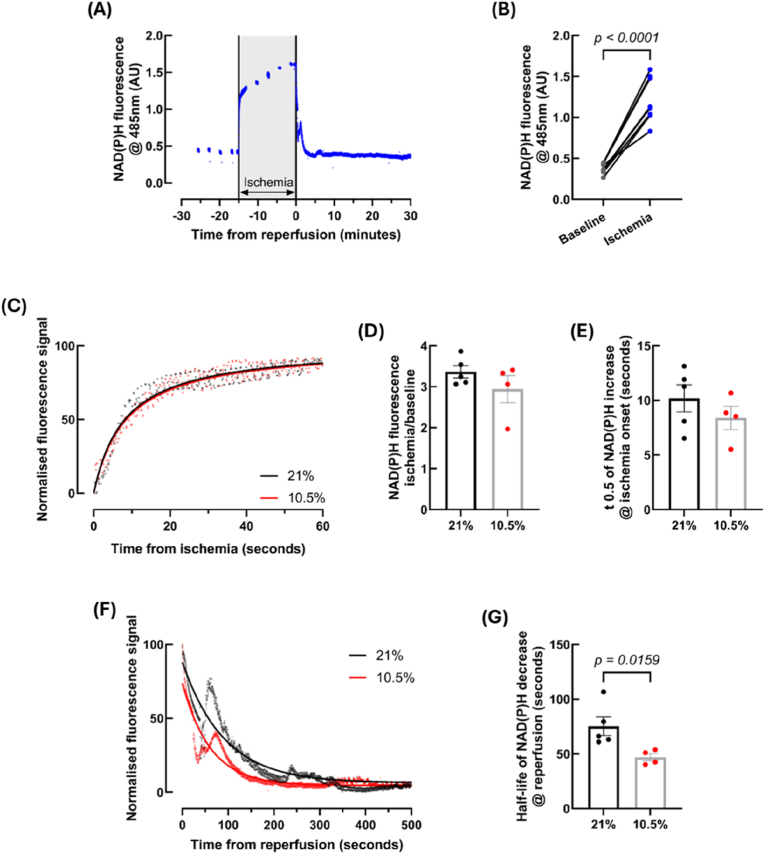
**Adult rats subject to fetal hypoxia (GD 15**–**20, 10.5 % O_2_) show altered NAD(P)H metabolism at reperfusion following ischemia.** NAD(P)H autofluorescence demonstrated the expected fluorescence pattern at 485 nm when subject to ischemia-reperfusion (A), showing a roughly 3-fold increase in fluorescence from baseline to ischemia (B). Representative traces show the initial increase in fluorescence at ischemia (C), which was used to calculate fluorescent fold change from baseline to ischemia (D) and the t = 0.5 of NAD(P)H fluorescence increase (E). Representative traces show the decrease in fluorescent signal upon reperfusion (F), used to calculate the half-life of NAD(P)H fluorescence decrease (G). *n = 5 (21 %) and 4 (10.5 %). NAD(P)H = nicotinamide adenine dinucleotide phosphate hydrogen. Data is presented as mean ± SEM. Significance was tested using a paired two-tailed t-test for (B), and Mann-Whitney tests for (D), (E) and (G).*

Heart NAD(P)H fluorescence during IR displayed the expected pattern, whereby fluorescence emission at 485 nm rapidly increased when the tissue became ischemic (NAD(P)H reduction) and decreased to baseline fluorescence at reperfusion (NAD(P)H oxidised to NAD^+^) (Fig. 8A and B). No differences were observed between the experimental groups for the rate at which fluorescence increased upon ischemia onset when NAD(P)H was reduced (Fig. 8C and E). Additionally, the fold change of signal from ischemia to baseline was comparable between the two groups (Fig. 8D). However, the hypoxic group demonstrated a faster rate of NAD(P)H oxidation during the reperfusion period, shown by a reduced half-life of the normalised signal (*p* = 0.0159) (Fig. 8F and G).

**Fig. 9:**
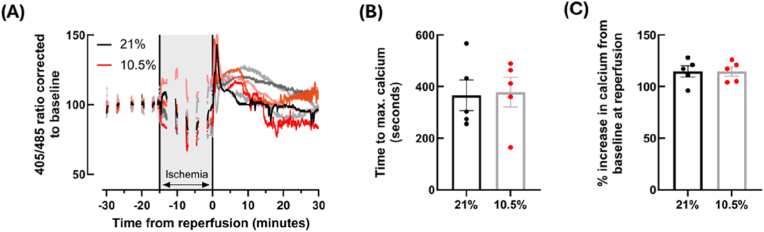
**Calcium increases at reperfusion, but no differences are observed between control (21 % O_2_) and hypoxic (GD 15**–**20, 10.5 % O_2_) hearts.** Timeline of 405/485 ratio across washout, ischemia and reperfusion, with 21 % recordings shown in black/grey and 10.5 % in red/pink – all values were normalised to their baseline value taken at the end of the washout period, directly prior to ischemia (A). The time taken to reach the maximum calcium value during reperfusion (excluding the initial spike in fluorescence at reperfusion) (B) and the percentage increase from the end of baseline to the maximum calcium value during reperfusion (C) were calculated. *n = 5 (21 %) and 5 (10.5 %). Data is presented as mean ± SEM. Significance was tested using two-tailed t-tests for (B) and (D).*

Calcium transients were successfully measured in *ex vivo* hearts by Indo-1 fluorescence (Supplementary Figure 6E). With the exception of one animal, intracellular calcium decreased during the initial ischemic period, and then slowly increased for the remainder of ischemia. Intracellular calcium consistently increased from baseline during reperfusion, as attested by the increased 405/485 nm ratio (Fig. 9A). However, this increase was comparable between experimental groups, as demonstrated by similar values for the time to peak calcium after reperfusion (Fig. 9B), and percentage change in calcium from baseline to reperfusion (Fig. 9C). Collectively, these results suggest there is no difference in the mean cardiac myocyte calcium handling during IR between offspring from hypoxic pregnancies and controls**.**

**Fig. 10:**
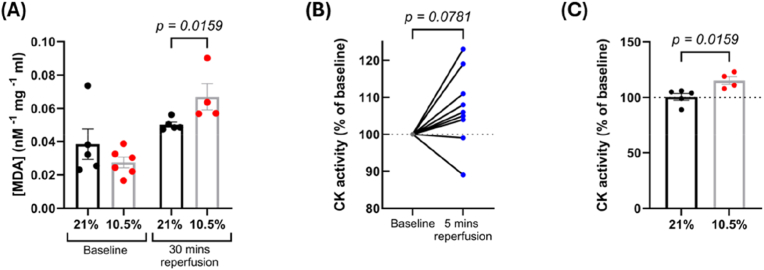
**Adult rats subjected to fetal hypoxia (GD 15**–**20, 10.5 % O_2_) show increased oxidative stress and creatine kinase activity after ischemia-reperfusion.** Malondialdehyde (a marker of lipid peroxidation) was measured in heart ventricular tissue using a TBARS assay, either in baseline tissue (not exposed to IR), or following 15 min ischemia and 30 min reperfusion, and normalised to protein content (A). CK activity was measured in effluent collected at baseline and 5 min post reperfusion, normalised to baseline activity (B) and compared between experimental groups (C). *n = 5 (21 %) and 4–6 (10.5 %). MDA = malondialdehyde, CK = creatine kinase. Data is presented as mean ± SEM. Significance was tested using Mann-Whitney tests for (A) and (C), and a Wilcoxon test for (B)*.

No differences were observed between the groups in baseline levels of oxidative stress (assessed by malondialdehyde lipid peroxidation) in ventricular tissue that was not subjected to IR (Fig. 10A). However, oxidative stress was significantly higher in the hypoxic group after IR, compared to post-IR controls (*p* = 0.0159) (Fig. 10A). CK activity across both groups tended to be increased at reperfusion compared to baseline (*p* = 0.0781) (Fig. 10B), with activity in the hypoxic group increasing more than the controls at reperfusion (*p* = 0.0159) (Fig. 10C)**.**

## 4 Discussion

Our study shows, for the first time, that the MPTP pathway can be programmed by intrauterine stress. Specifically, we found that fetal hypoxia increases MPTP calcium sensitivity, and this is associated with increased protein abundance of MPTP components, enhanced mitochondrial H_2_O_2_ emission, increased sensitivity to oxidative stress and tissue damage, and altered ETS activity. Taken together, these results help to explain why offspring from hypoxic pregnancies have poor post-ischemic ventricular recovery. The findings have clinical implications for children from hypoxic pregnancies, who are at a higher risk of ischemic heart disease and myocardial infarction.

### 4.1 MPTP calcium sensitivity is increased by fetal hypoxia

The major finding from this study is that fetal hypoxia (10.5 % oxygen) increases MPTP calcium sensitivity in adult offspring (a decrease in CRC). This demonstrates that the intrinsic properties of the MPTP can be programmed by the intrauterine environment. Indeed, we found that protein abundance of the MPTP component CypD was increased in adult offspring from hypoxic pregnancies, which is known to heighten MPTP calcium sensitivity [31,[55], [56], [57], [58]], and impair IR recovery [31,59,60]. A previous study using a similar experimental model found that fetal hypoxia (10.5 % O_2_, GD 15-21) leads to differential methylation of the *Ppif* gene that encodes CypD [38], implicating epigenetic modification. However, contrary to both our mRNA and protein results, *Ppif* was downregulated in adults from hypoxic pregnancies [38]. This difference could be attributed to the methodology used, as the study by Huang et al. [38] utilised RNA-seq, whereas the present study used qPCR. The two methodologies demonstrate discordance in the region of 20 % [61], with RNA-seq more susceptible to false positives and qPCR often required to validate any significant expression changes reported following RNA-seq [62,63].

The discrepancy between the mRNA and protein results could be explained by the tissue type used for each analysis. Whilst qPCR was carried out on homogenised left ventricular tissue, all adult protein analysis was performed on isolated mitochondria. Therefore, the enrichment of mitochondrial proteins (i.e. CypD) prior to protein quantification would have increased the sensitivity, allowing detection of more subtle changes in protein abundance [64].

Another factor to consider is the presence of post-translational modifications (PTMs). PTMs not only influence degradation and protein half-life [[65], [66], [67]], but they are also implicated in disease and can affect MPTP opening [65,66,68,69]. In this regard, previous work has shown the expression and activity of one of the major post-translational modifiers of CypD, sirtuin 3 [70], is increased in fetal hearts during hypoxic pregnancy [71], which may explain the increased protein abundance of CypD in our study.

In addition to CypD abundance, we also measured protein expression of another putative MPTP component, the ATP synthase ring-forming c subunit and α subunit. Although it remains controversial, recent evidence suggests the c subunit can expand when matrix calcium concentration is high, leading to pore opening in the outer mitochondrial membrane [[72], [73], [74]]. We found no change in the abundance of these subunits in adults, but the c subunit was increased in neonatal hearts. Similar results have been published elsewhere [75], and suggest that subunits of the ATP synthase are upregulated as a direct result of fetal hypoxia, possibly to compensate for the lower O_2_ availability. However, our data suggests expression levels return to control levels at some stage between neonatal day 1 and adulthood.

### 4.2 IR sensitivity in offspring from hypoxic pregnancies was associated with increased oxidative stress and tissue damage, but not intracellular calcium concentration

Calcium is the main activator of the MPTP, and our previous work has shown that fetal hypoxia programmes abnormalities in cardiac myocyte calcium handling [37]. This phenotype is particularly dangerous during reperfusion, because the calcium that accumulates during ischemia will be more difficult to remove, leading to a higher probability of MPTP opening. Therefore, we hypothesised that offspring from hypoxic pregnancies would have higher levels of intracellular calcium during reperfusion, compared to controls. Similar to previous work (Supplementary Table 3), we showed that adult male offspring from hypoxic pregnancies were more sensitive to IR than controls, as attested by elevated LVEDP, increased oxidative stress and heightened CK activity at reperfusion. However, while we consistently observed an increase in intracellular calcium at reperfusion (which is thought to coincide with MPTP opening [52]), the magnitude and kinetics of the rise in calcium was comparable between the experimental groups. This is despite research from our group that show calcium kinetics at the individual myocyte level are altered in adult offspring following fetal hypoxia, demonstrating reduced speed of calcium uptake and impaired diastolic recovery of calcium [37]. Therefore, we suspect that our approach was not sensitive enough to determine subtle differences in calcium dynamics that may have occurred either in systole or diastole.

Instead, we found that rats from hypoxic pregnancies had a faster rate of NAD(P)H oxidation at reperfusion, demonstrated by a reduced half-life of NAD(P)H fluorescence. An accelerated rate of NAD(P)H oxidation has previously been associated with poor post-ischemic recovery [52,76], although the reasons for this are currently unclear. One hypothesis is a disrupted balance between NAD(P)H consumption and replenishment which may be the result of altered NAD(P)H oxidase (NOX) expression. Indeed, previous research has shown an increase in NOX2 expression in the aorta following fetal hypoxia with subsequent enhancement of ROS generation [77], which could help explain the increased oxidative stress observed in this study. Furthermore, efforts to maintain matrix NADH at reperfusion (e.g. inhibition of complex I) can improve IR injury by favouring NAD(P)H production and ROS detoxification [78]. Therefore, our results suggest increased rates of NAD(P)H oxidation may contribute to IR injury in offspring from hypoxic pregnancies.

### 4.3 Moderate fetal hypoxia increases mitochondrial H_2_O_2_ emission

ROS increase dramatically during reperfusion, and this contributes to myocardial injury by modulating the calcium sensitivity of the MPTP and increasing the open probability [79]. Given that our previous work has shown fetal hypoxia increases basal ROS production across the life course [36,53], we hypothesised that offspring from hypoxic pregnancies would have greater ROS production during reperfusion. Indeed, in accordance with previous studies [36,80], animals from hypoxic pregnancies had higher basal H_2_O_2_ emission and greater levels of oxidative stress than controls during reperfusion, despite comparable levels prior to ischemia. Moreover, we observed that in the presence of antimycin A, H_2_O_2_ emission was higher in the 10.5 % hypoxic group versus controls. Under these experimental conditions, H_2_O_2_ emission mainly occurs at the complex III-Qo site, which is known to be a major source of oxidative stress in the postischemic heart [81,82]. These results support our earlier findings in a mouse model of fetal hypoxia (14 % O_2_, GD 6–18) [36], suggesting that increased basal H_2_O_2_ in adulthood may be a common feature of offspring hearts from hypoxic pregnancies. Additionally, previous research has shown that animals exposed to fetal hypoxia exhibit lower abundance of CIII subunits [39,75], which could also contribute to ROS emission through decreased electron transfer efficiency. Previous work has shown higher basal levels of H_2_O_2_ triggers greater downstream ROS release (either through MPTP opening or reduced efficiency of electron transport through the ETS), via ROS-induced ROS release [34,83]. This phenomenon may be sufficient to induce oxidative stress after IR in offspring from hypoxic pregnancies.

### 4.4 Electron transport system activity is altered by moderate fetal hypoxia

Similar to previous work [36], we observed a reduction in respiratory flux through CIV of the ETS in the 10.5 % hypoxic group. This effect has been reported in other models and can be driven by a reduced CIV content or activity [36,40,84]. A reduction in CIV could worsen IR injury by reducing the aerobic capacity of mitochondria during reperfusion [85,86]. Furthermore, a role for CIV activity in MPTP activation has previously been proposed [87,88], with a reduction in CIV activity correlating with increased sensitisation to apoptotic signals [89]. Although suggestive of a decrease in CIV content or activity, the reduction in respiratory flux observed here is unlikely to have a major impact on overall mitochondrial respiratory rate *in vivo.* This is due to the “biochemical threshold effect” [90], whereby CIV inhibition has to exceed 75 % before a decrease in mitochondrial respiration is observed *in vivo*, due to the high abundance of CIV [91].

Despite the reduction in CIV activity, we found that fetal hypoxia increased O_2_ affinity. This may represent a compensatory response to offset mitochondrial dysfunction, which has been previously demonstrated in animals living at high-altitude [92]. The regulation of CIV is dependent on expression of subunit isoforms [93,94], and therefore it is possible that fetal hypoxia alters the expression of CIV isoforms to increase O_2_ affinity, at the expense of reduced oxidative capacity. In support of this hypothesis, hypoxia is known to induce isoform switching of cytochrome *c* oxidase (COX) 4–1 to COX 4–2 through HIF-1α signalling [95]. COX4-2 has been associated with reduced O_2_ consumption rates and increased superoxide production [96,97] which align with this study. However, the O_2_ affinity of COX4-2 is lower than its COX4-1 counterpart [93]. Clearly, more work is required to determine the relative expression of each COX isoform in the heart following fetal hypoxia and the subsequent effect on cardiac function.

### 4.5 Comparison of the 10.5 % and 13 % hypoxia models

As expected, mitochondrial outcomes in offspring from the 10.5 % hypoxic group were worse than the milder 13 % hypoxic insult. Indeed, the only significant difference observed between the 13 % hypoxic group and control was an increased abundance of CypD. Importantly, the neonatal pups from the 10.5 % O_2_ pregnancies tended to weigh less than controls, demonstrating a degree of FGR that commonly occurs in hypoxia [9,[98], [99], [100]]. The FGR may have resulted from the observed decrease in maternal food intake, or the direct effect of hypoxia. Nevertheless, the FGR was mild (∼10 % smaller than controls), suggesting that mitochondrial dysfunction likely occurred in response to hypoxia, rather than fetal undernutrition. Clinically, the results presented here suggest that offspring from pregnancies that are complicated by late-onset hypoxia (e.g. pre-eclampsia) may have greater sensitivity to cardiac conditions (such as myocardial infarction) than early-onset hypoxic pregnancies. It is important to take these factors into consideration when designing maternal therapeutics such as the use of antenatal antioxidants or maternal oxygen supplementation.

## 5 Limitations

One limitation of this study is the absence of female offspring. Previous work has shown limited effects of fetal hypoxia on female adult offspring compared to male offspring with regards to IR injury, with the majority of research reporting no significant changes from normoxic hearts following IR [18,20,101,102]. However, sex differences in MPTP sensitivity to calcium have been reported [103], and various groups have demonstrated similar sensitivity to IR between males and females following fetal hypoxia [20,104], so investigating potential programming of the MPTP in female offspring would be prudent and should be addressed in future experiments. We were also limited by the inability to measure discrete systolic and diastolic calcium levels, as well as the corresponding speed of calcium release and reuptake, in the whole heart during our IR experiments, as this required a higher temporal resolution and higher tissue light exposure that would quickly photo-bleach the fluorescent dye and promote photo-toxicity in the heart tissue. Therefore, any subtle changes to calcium handling and dynamics that may have occurred in the heart following fetal hypoxia would have been missed. Finally, all whole heart experiments were only performed using the control and 10.5 % hypoxia groups, with no data collected from the 13 % hypoxia group for these experiments. This decision was made as the altered mitochondrial phenotype was only observed in the 10.5 % group. However, determining IR sensitivity and the associated mechanistic data in the 13 % group would have been useful to further assess the relationship between the mitochondrial phenotype and corresponding IR sensitivity.

## 6 Conclusion and future perspectives

Our study demonstrates that fetal hypoxia can programme MPTP calcium sensitivity. This was associated with changes in MPTP modulators, including CypD expression, NAD(P)H levels, basal ROS emission and ETS activity. Together, this may help to explain the decreased post-ischemic recovery commonly seen in offspring from hypoxic pregnancies. Future studies could expose CypD knockout mice to fetal hypoxia to further confirm the MPTP's role in programming IR sensitivity. It would also be interesting to directly measure mitochondrial calcium with mitochondrially-targeted fluorescent probes. Although we did not find any differences between experimental groups in calcium management during reperfusion, it is still possible that mitochondrial calcium levels were higher in offspring from hypoxic pregnancies.

Collectively, our findings support the notion that mitochondria are a key target of developmental programming and provides additional evidence to help explain the increased IR sensitivity in offspring from hypoxic pregnancies. Further studies on the underlying molecular mechanisms may help to develop future maternal therapeutics to prevent cardiac programming. Given the fundamental role of the MPTP in programmed cell death, intrauterine programming of this pathway has significant implications beyond cardiovascular disease. Indeed, the MPTP is a ubiquitous initiator of cell death that has been implicated in a wide range of diseases including metabolic and neurodegenerative disorders [[105], [106], [107], [108]]. Therefore, MPTP programming likely plays an important role in a large number of diseases with a developmental origin.

## CRediT authorship contribution statement

**Kerri L.M. Smith:** Conceptualization, Data curation, Formal analysis, Investigation, Methodology, Writing – original draft, Writing – review & editing. **Philippe Pasdois:** Data curation, Investigation, Methodology, Writing – review & editing. **Mafalda Pires:** Investigation, Writing – review & editing. **Mitchell C. Lock:** Data curation, Investigation, Writing – review & editing. **Gina L.J. Galli:** Funding acquisition, Methodology, Supervision, Writing – review & editing.

## Declaration of competing interest

The authors declare that they have no known competing financial interests or personal relationships that could have appeared to influence the work reported in this paper.
