# Supplementary material for: Fetal programming of the cardiac mitochondrial permeability transition pore in male offspring from hypoxic pregnancies
Source: Redox Biol. 2025 Dec 11;89:103975. doi: 10.1016/j.redox.2025.103975 (PMC12814092; doi:10.1016/j.redox.2025.103975)
Supplement: Multimedia component 1 [file mmc1.docx]

# Supplementary

| **Primer name** | **Target gene** | **Primer sequence 5’ to 3’** | **Accession number** |
| --- | --- | --- | --- |
| ***Rpl32*** | Ribosomal protein L32 | F – GCCCAAGATCGTCAAAAAGA  R - AATCTTCTCCGCACCCTGTT | NM_013226.3 |
| ***Rpl4*** | Ribosomal protein L4 | F – GCCAAATCGGAGAAGATTGT  R - TGCAGGCTTCTTCAGCTTCT | BC081801.1 |
| ***Polr2a*** | RNA polymerase II subunit A | F – TCTCCCACTTCTCCTGGCTA  R - CTCCTCATCGCTGTCTTCTG | NM_001427041.1 |
| ***Atp5f1a*** | ATP synthase F1 subunit alpha | F – GTCCGCCTACATTCCAACAA  R - GACACAGACAAGCCCACATTA | NM_023093.1 |
| ***Atp5mc1*** | ATP synthase membrane subunit c locus 1 | F – AGACCAGAGGCTCCATCTAA  R - CCAATAAACTTGGCTGCTGTG | NM_017311.1 |
| ***Ppif*** | Peptidyl-prolyl isomerase F | F – TCTTGTGAAAGTCCCACCATC  R - CAGGTCCAGCTGCTTCTATTT | NM_172243.2 |

**Supplementary Table 1. Primer sequences for qPCR.**

| **Experimental group** | **n** | **% increase with cytochrome c** | **RCR (State 3:State 4 w/oligo)** | **P/E ratio** | **Citrate synthase activity** |
| --- | --- | --- | --- | --- | --- |
| **Control (21%)** | 8 | 13% ± 1% | 6.7 ± 0.5 | 0.82 ± 0.02 | 1.08 ± 0.05 |
| **Hypoxic (13%)** | 7 | 11% ± 1% | 6.4 ± 0.4 | 0.83 ± 0.02 | 1.09 ± 0.05 |
| **Hypoxic (10.5%)** | 7 | 13% ± 1% | 6.9 ± 0.3 | 0.84 ± 0.02 | 1.05 ± 0.04 |

**Supplementary Table 2. Mitochondrial quality and efficiency parameters.** State 3 as ADP-stimulated respiration (CI substrates), state 4 as leak with adenylates following oligomycin inhibition, P as ADP-stimulated respiration (CI+CII substrates), E as electron transport capacity (uncoupled with FCCP). *Units for citrate synthase activity are (OD min^-1^ mg^-1^). Data is presented as mean ± SEM. Significance was tested using one-way ANOVA for all parameters except* % increase with cytochrome c *which used Kruskal-Wallis.*

| **Reference** | **Animal model** | **Term** | **Hypoxia challenge** | **Sex** | **FGR** | **Age @ IR** | **Set up** |  |
| --- | --- | --- | --- | --- | --- | --- | --- | --- |
| Li et al, J Soc Gynecol Investig, 2003 | Sprague-Dawley rats | 22 days | 10.5% O₂ GD15-21 | Male | Yes (4.87g vs 6.28g) | 6 months | *ex vivo*, Langendorff |  |
|  |  |  |  |  |  |  |  |  |
| Zhang et al, Int J Cardiol, 2019 | Sheep (mixed Western breed) | ~140 days | ~13% O₂ GD30-140* | Male | No | Near-term fetus | *ex vivo*, Langendorff |  |
|  |  |  |  | Female | Yes (4.41kg vs 3.4kg) |  |  |  |
| Xue and Zhang, J Pharmacol Exp Ther, 2009 | Sprague-Dawley rats | 22 days | 10.5% O₂ GD15-21 | Male | Data not shown | 3 months | *ex vivo*, Langendorff |  |
|  |  |  |  | Female |  | 3 months |  |  |
| Niu et al, Hypertension, 2018 | Wistar rats | 21 days | 13% O_2_ GD6-20 | Male | No | 4 months | *ex vivo*, Langendorff |  |
| Xiong et al, J Mol Cell Cardiol, 2016 | Sprague-Dawley rats | 22 days | 10.5% O₂ GD15-21 | Mixed male and female | Data not shown | 4 weeks | *ex vivo*, Langendorff |  |

| **Reference** | **IR challenge** | **LVEDP** | **LVDP** | **RPP** | **LDH release** | **Coronary flow** |
| --- | --- | --- | --- | --- | --- | --- |
| Li et al, J Soc Gynecol Investig, 2003 | 10 minutes ischemia, 3 hours reperfusion | Increased | Decreased | Decreased | Not measured | Decreased |
|  | 25 minutes ischemia, 3 hours reperfusion | Increased | Decreased | Decreased | Not measured | Data not shown |
| Zhang et al, Int J Cardiol, 2019 | 20 minutes ischemia, 1 hour reperfusion | Increased | Decreased | Data not shown | Not measured | Decreased |
|  |  | Increased | Decreased | Data not shown | Not measured | Decreased |
| Xue and Zhang, J Pharmacol Exp Ther, 2009 | 20 minutes ischemia, 30 minutes reperfusion | Increased | Decreased | Data not shown | Increased | Unchanged |
|  |  | Unchanged | Unchanged | Data not shown | Unchanged | Data not shown |
| Niu et al, Hypertension, 2018 | 10 minutes ischemia, 30 minutes reperfusion | Data not shown | Decreased | Data not shown | Increased | Data not shown |
| Xiong et al, J Mol Cell Cardiol, 2016 | 45 minutes ischemia, 30 minutes reperfusion | Increased | Decreased | Data not shown | Increased | Not measured |

**Supplementary Table 3. Previous research demonstrating phenotype of increased sensitivity to IR following fetal hypoxia.** All measures shown are hypoxic group compared to normoxic. *All animals experienced hypoxia in an environmental chamber except for Zhang *et al* 2019, where hypoxia was experienced through altitude. The Pubmed search terms used were “ischemia reperfusion AND (heart OR cardiac) AND (developmental hypoxia OR fetal hypoxia OR prenatal hypoxia OR gestational hypoxia)”. Only research with relevant parameters to the current manuscript are included in this table; a more detailed table can be found on figshare (DOI 10.6084/m9.figshare.30800705) ^8,11-15,93-97^. *FGR = fetal growth restriction, IR = ischemia reperfusion, LVEDP = left ventricular end diastolic pressure, LVDP = left ventricular developed pressure, RPP = rate pressure product, LDH = lactate dehydrogenase.*

|  |  |  | **Time post reperfusion (minutes)** | | | | | | |
| --- | --- | --- | --- | --- | --- | --- | --- | --- | --- |
|  | **Experimental group** | **n** | **Baseline** | **5** | **10** | **15** | **20** | **25** | **30** |
| **RPP (raw values [mmHg/min])** | **Control (21%)** | 6 | 27817 ± 1745 | 22827 ± 4313 | 24225 ± 4422 | 29116 ± 2100 | 26995 ± 2618 | 27005 ± 2096 | 26163 ± 1642 |
|  | **Hypoxic (10.5%)** | 6 | 31350 ± 1121 | 26530 ± 3013 | 28538 ± 2845 | 28980 ± 2859 | 28925 ± 2503 | 28234 ± 2292 | 27448 ± 1858 |
| **RPP (% of baseline)** | **Control (21%)** | 6 | 100 | 82 ± 11 | 89 ± 17 | 109 ± 10 | 99 ± 11 | 98 ± 7 | 95 ± 4 |
|  | **Hypoxic (10.5%)** | 6 | 100 | 84 ± 10 | 91 ± 9 | 92 ± 9 | 92 ± 8 | 90 ± 6 | 87 ± 5 |
| **LVEDP (mmHg)** | **Control (21%)** | 6 | 7.45 ± 0.93 | 12.16 ± 4.22 | 11.95 ± 2.49 | 12.36 ± 2.91 | 11.79 ± 2.47 | 11.02 ± 2.53 | 11.08 ± 2.51 |
|  | **Hypoxic (10.5%)** | 6 | 8.74 ± 0.68 | 20.16 ± 2.66 | 20.01 ± 2.89 | 17.41 ± 2.36 | 16.80 ± 2.33 | 16.73 ± 2.6 | 16.52 ± 2.54 |

**Supplementary Table 4. Functional parameters at baseline and post IR in rats from control (21% O_2_) and hypoxic (10.5% O_2_, GD15-20) pregnancies.** Values were taken at 5- and 10-minutes pre-ischemia and averaged to obtain the baseline values, and then at 5-minute intervals after reperfusion of the heart. *RPP = rate pressure product, LVEDP = left ventricular end developed pressure. Significance was tested using a Mann-Whitney test.*


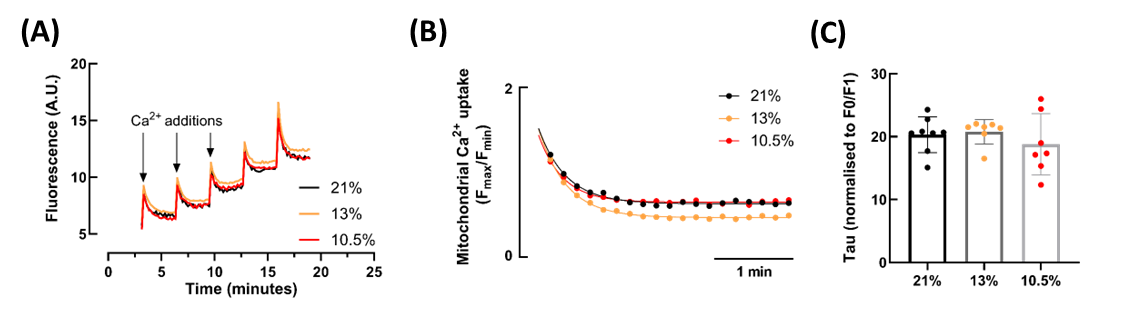


**Supplementary Figure 1. Mitochondrial calcium uptake to measure activity of the MCU.** Enlarged portion of traces from Figure 5A showing calcium additions 2 – 6 (A), which were normalised (B) and used to calculate *tau* values (averaged for each animal from addition 2 – 6) (C). Significance was tested using a Kruskal-Wallis test.


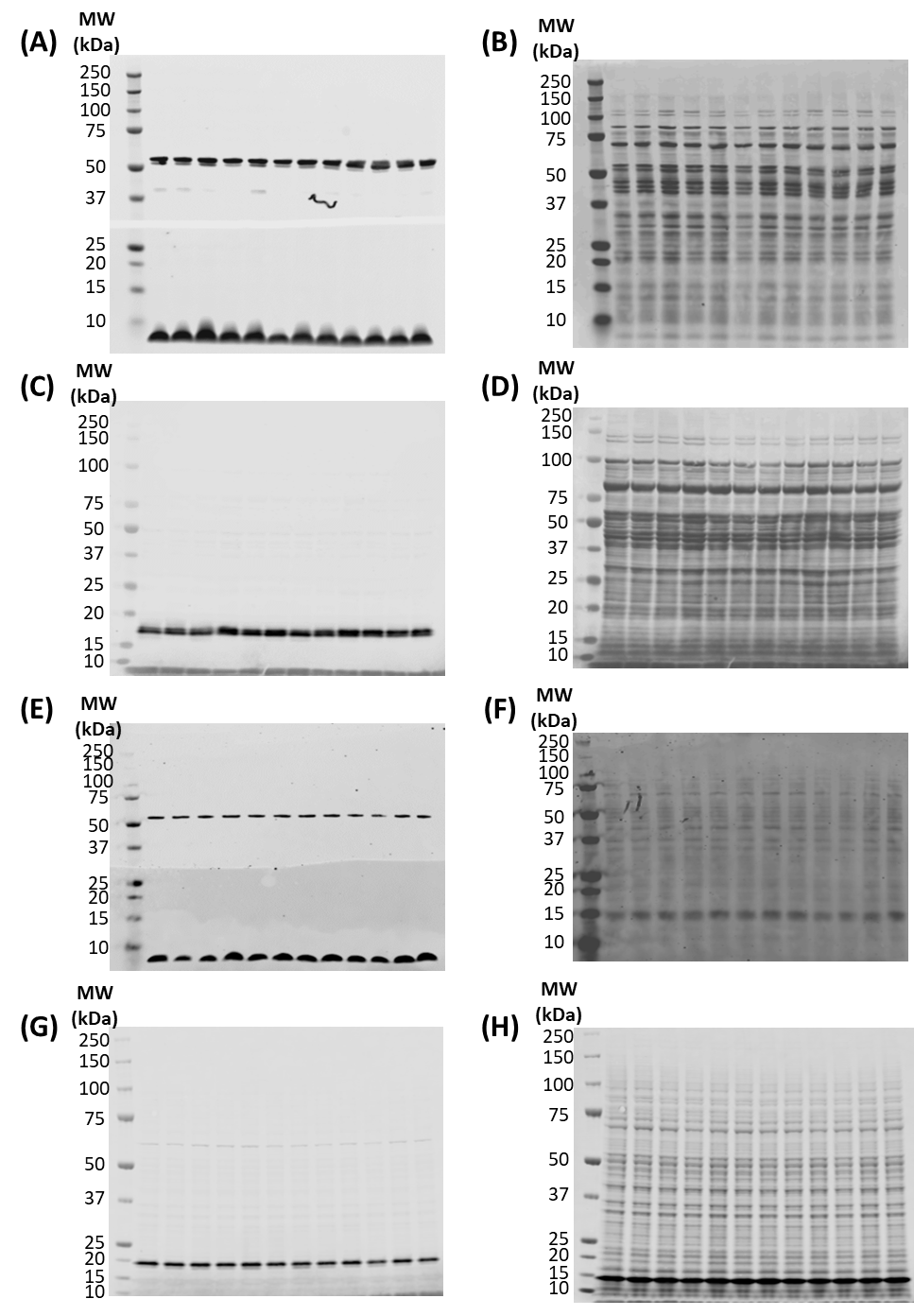


Supplementary Figure 2. Raw Western blot images. Adult ATP synthase α and c subunit bands (A) and total protein visualisation (B). Adult CypD band (C) and total protein visualisation (D). Neonatal ATP synthase α and c subunit bands (E) and total protein visualisation (F). Neonatal CypD band (G) and total protein visualisation (H).


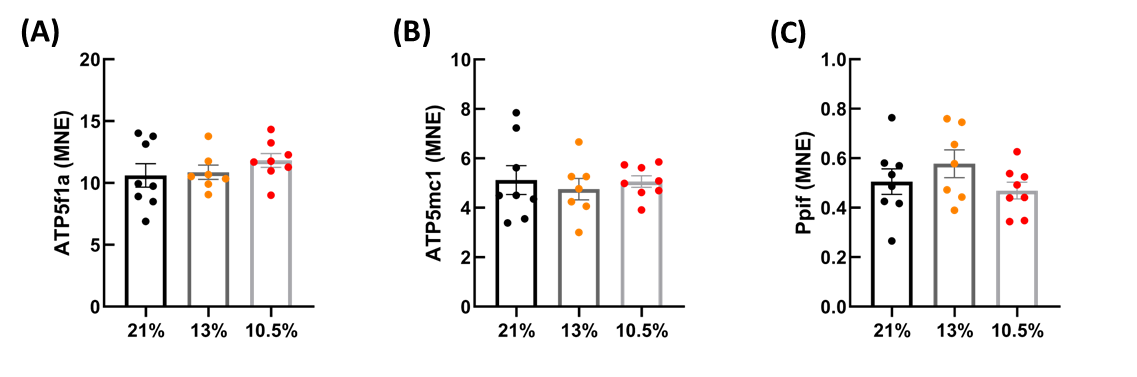


**Supplementary Figure 3. mRNA expression of components of the mitochondrial permeability transition pore.** mRNA expression of *ATP5f1a* (A), *ATP5mc1* (B) and *Ppif* (C). Expression was normalised to housekeeper genes *Rpl32, Rpl4* and *Polr2a*. *MNE = mean normalised expression. Significance was tested using one-way ANOVA.*


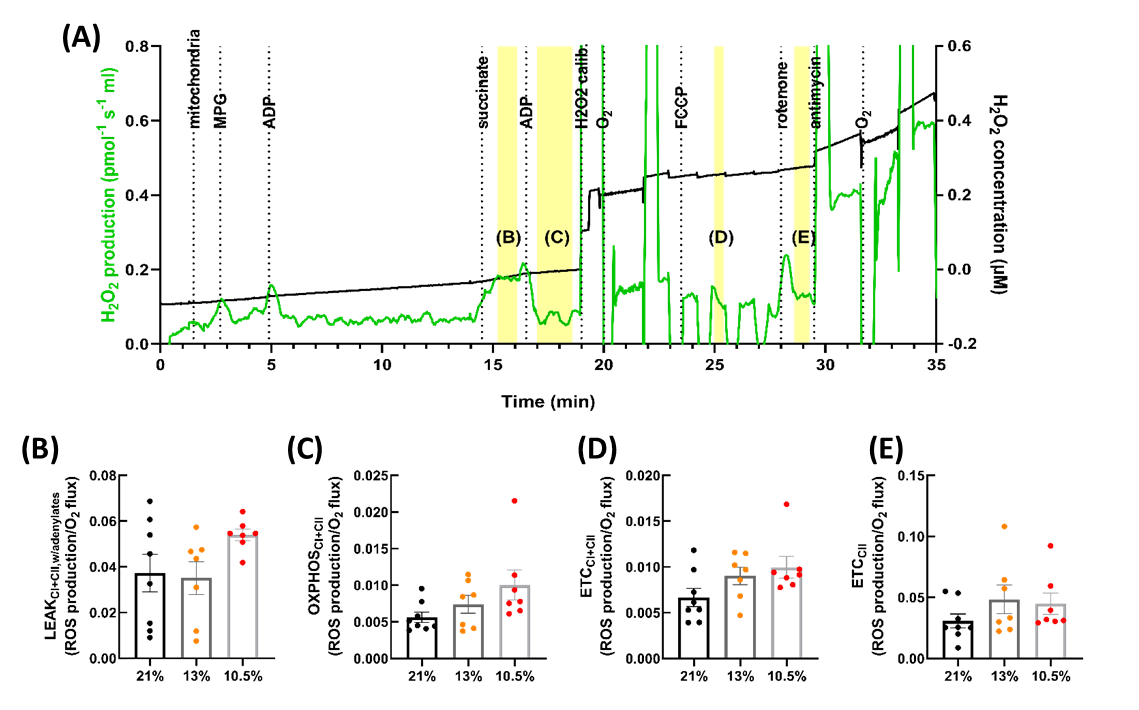


**Supplementary Figure 4. Original trace of hydrogen peroxide (H₂O₂) emission.** H₂O₂ emission (A) of isolated mitochondria from an adult rat exposed to fetal hypoxia (10.5% O₂, GD15-20). Prior to mitochondria injection, horseradish peroxide, Amplex UltraRed and superoxide dismutase were added in order to measure H₂O₂ emission. Compounds were added sequentially to measure H₂O₂ emission at each complex of the electron transport system. Measurements were taken from a stable region of the trace, once injection artefacts had stabilised. Each panel shows a different respiratory state, that relates to the highlighted region on the example Oroboros trace, as follows: Leak respiration with CI and CII substrates, with adenylates (B), oxidative phosphorylation with CI and CII substrates (C), electron transfer capacity with substrates for CI and CII (D), electron transfer capacity with substrates for CII (E). *MPG = malate, pyruvate and glutamate, ADP = adenosine diphosphate, FCCP = carbonyl cyanide-4-(trifluoromethoxy)phenylhydrazone, TMPD = N,N,N,N-tetramethyl-p-phenylenediamine.* *Significance was tested using Kruskal-Wallis for all parameters except for (B) which used one-way ANOVA.*

**
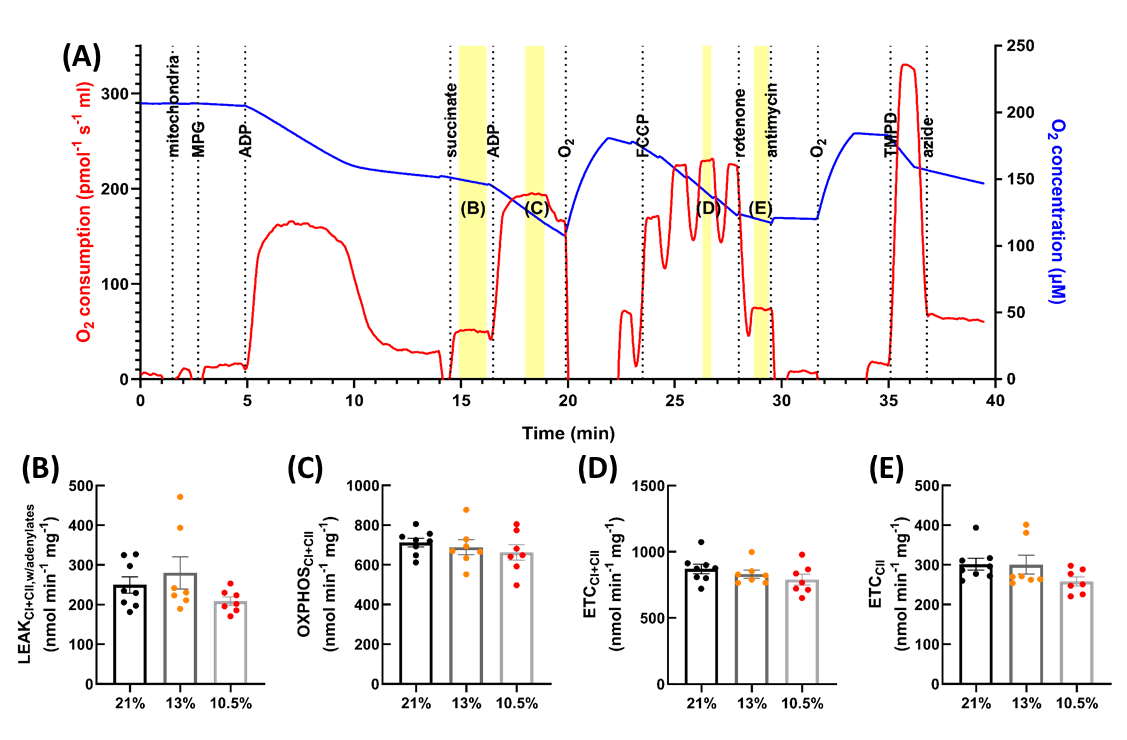
**

**Supplementary Figure 5. Original trace of mitochondrial oxygen consumption.** Mitochondrial O₂ consumption (A) measured in isolated mitochondria from an adult rat exposed to fetal hypoxia (10.5% O₂, GD15-20). Compounds were added sequentially to measure O₂ consumption at each complex of the electron transport system. Measurements were taken from a stable region of the trace, once injection artefacts had stabilised. Each panel shows a different respiratory state, that relates to the highlighted region on the example Oroboros trace, as follows: leak respiration with CI and CII substrates, with adenylates (B), oxidative phosphorylation with CI and CII substrates (C), electron transfer capacity with substrates for CI and CII (D) and electron transfer capacity with substrates for CII (E). *MPG = malate, pyruvate and glutamate, ADP = adenosine diphosphate, FCCP = carbonyl cyanide-4-(trifluoromethoxy)phenylhydrazone, TMPD = N,N,N,N-tetramethyl-p-phenylenediamine.* *Significance was tested using one-way ANOVA for all parameters except for (B) and (E) which used Kruskal-Wallis.*


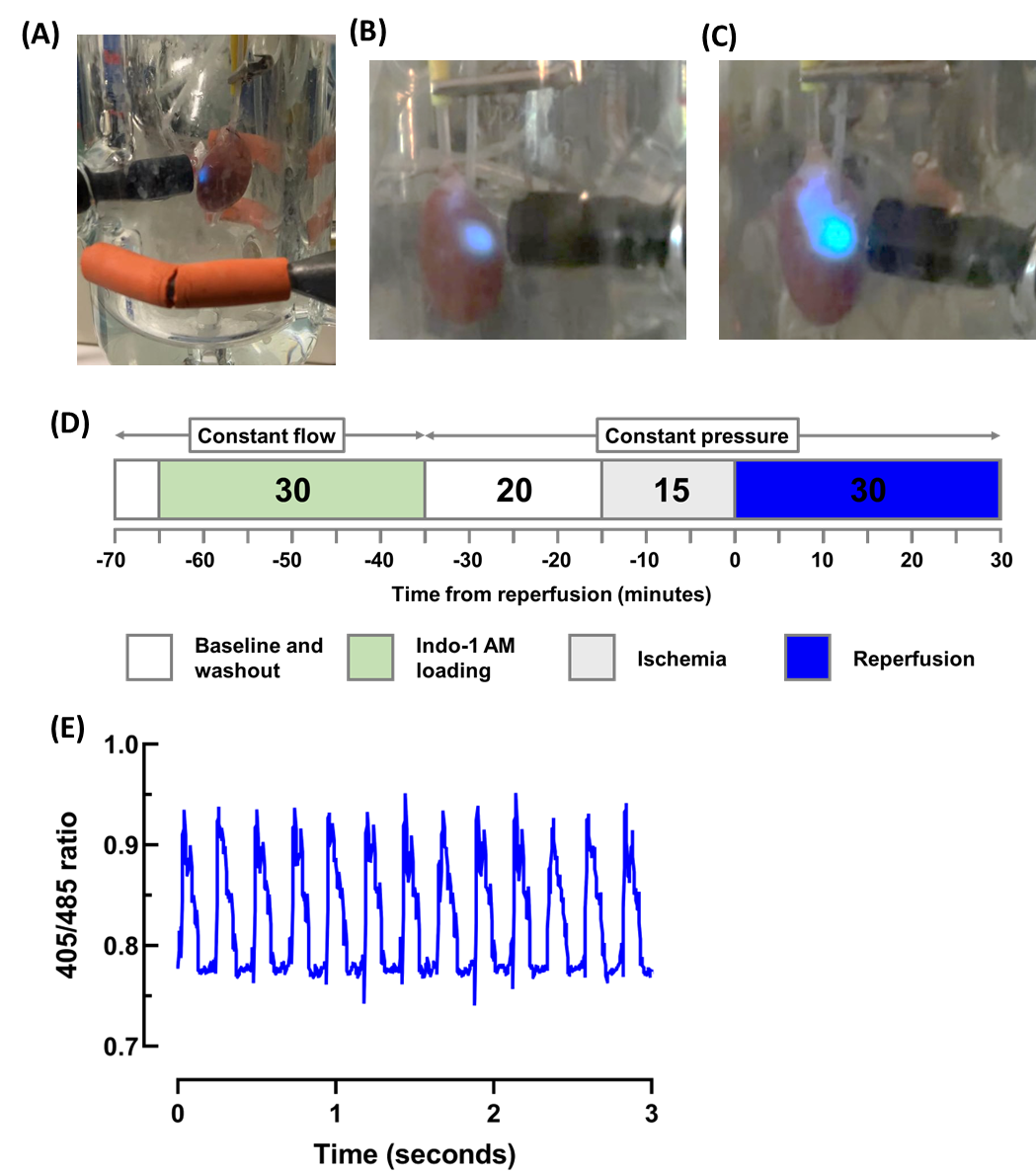


Supplementary Figure 6. Experimental set up for epicardial fluorescence measurements and loading protocol. Fluorescence was measured from the left ventricular free wall, with the LED pen positioned roughly 5mm away from the heart surface (A). The same heart before (B) and after (C) loading of Indo-1 AM (illumination at 340 nm). The Indo-1 AM loading protocol is detailed in (D). Calcium transients recorded after successful Indo-1 AM loading are shown in (E).


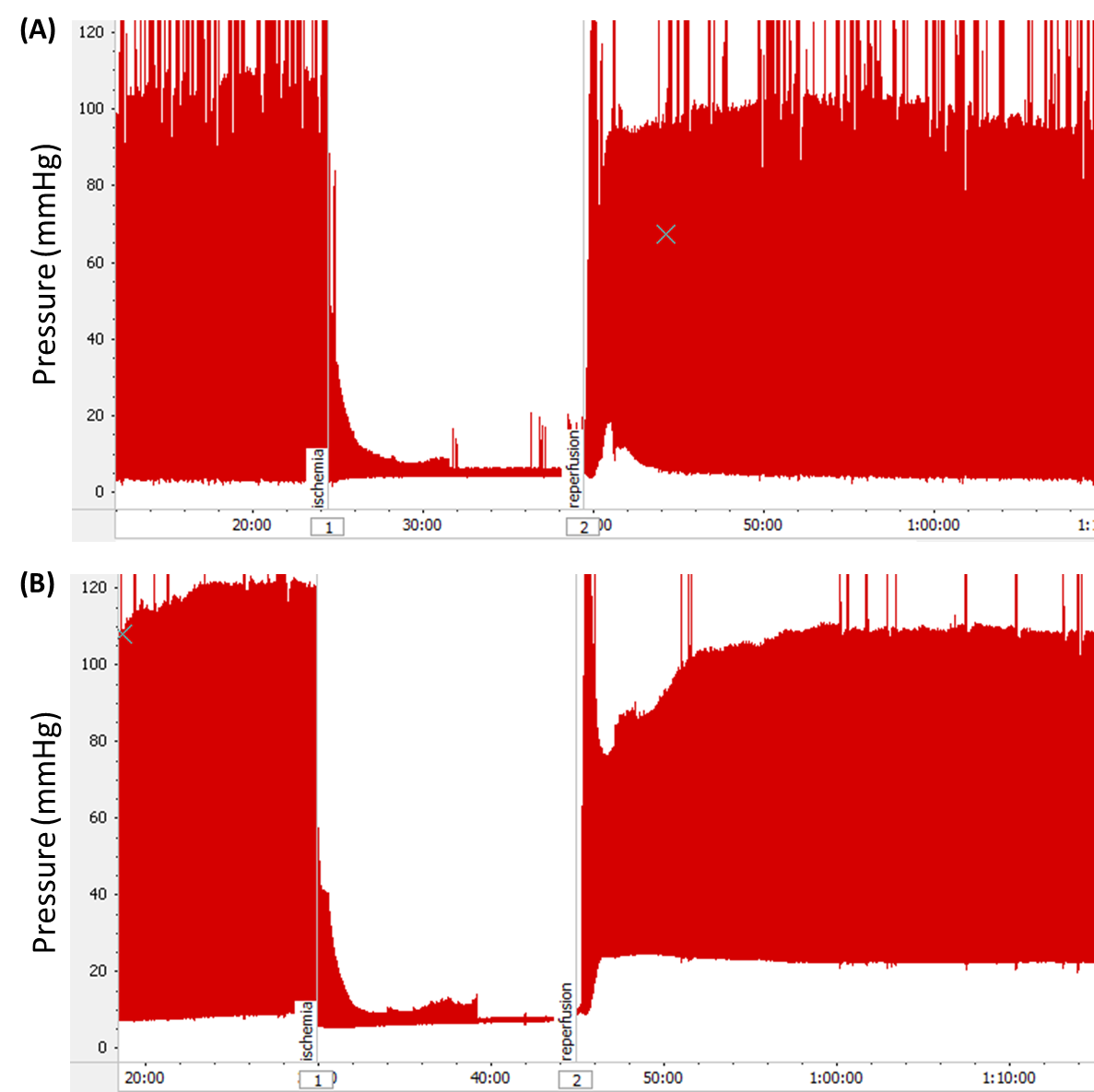


Supplementary Figure 7. Original trace of left ventricular pressure, LabChart v8, AD Instruments). Trace from a rat heart subject to (A) normoxic (21% O_2_) development and (B) hypoxic (10.5% O₂, GD15-20) development. The periods of ischemia and reperfusion are marked on the trace. Baseline measurements were taken at 5- and 10- minutes before ischemia, and subsequent measurements were taken at 5-minute intervals post reperfusion.
